# Deletion of endothelial leptin receptors in mice promotes diet-induced obesity

**DOI:** 10.1038/s41598-023-35281-7

**Published:** 2023-05-22

**Authors:** Rajinikanth Gogiraju, Claudius Witzler, Fatemeh Shahneh, Astrid Hubert, Luisa Renner, Magdalena L. Bochenek, Konstantinos Zifkos, Christian Becker, Madhusudhan Thati, Katrin Schäfer

**Affiliations:** 1 Department of Cardiology, Cardiology I, University Medical Center of the Johannes Gutenberg University Mainz, Mainz, Germany; 2 Center for Thrombosis and Hemostasis, University Medical Center Mainz, Mainz, Germany; 3 Clinic of Dermatology, University Clinic Münster, Münster, Germany

**Keywords:** Cardiovascular biology, Cell signalling, Cardiovascular biology, Metabolism, Cell biology, Physiology, Cardiology, Molecular medicine

## Abstract

Obesity promotes endothelial dysfunction. Endothelial cells not only respond, but possibly actively promote the development of obesity and metabolic dysfunction. Our aim was to characterize the role of endothelial leptin receptors (LepR) for endothelial and whole body metabolism and diet-induced obesity. Mice with tamoxifen-inducible, Tie2.Cre-ER^T2^-mediated deletion of LepR in endothelial cells (End.LepR knockout, KO) were fed high-fat diet (HFD) for 16 weeks. Body weight gain, serum leptin levels, visceral adiposity and adipose tissue inflammation were more pronounced in obese End.LepR-KO mice, whereas fasting serum glucose and insulin levels or the extent of hepatic steatosis did not differ. Reduced brain endothelial transcytosis of exogenous leptin, increased food intake and total energy balance were observed in End.LepR-KO mice and accompanied by brain perivascular macrophage accumulation, whereas physical activity, energy expenditure and respiratory exchange rates did not differ. Metabolic flux analysis revealed no changes in the bioenergetic profile of endothelial cells from brain or visceral adipose tissue, but higher glycolysis and mitochondrial respiration rates in those isolated from lungs. Our findings support a role for endothelial LepRs in the transport of leptin into the brain and neuronal control of food intake, and also suggest organ-specific changes in endothelial cell, but not whole-body metabolism.

## Introduction

Obesity is an established cardiovascular risk factor and frequently associated with endothelial dysfunction^1^, one of the earliest signs of vascular disease. The detrimental effects of obesity are mediated by a number of factors, including the chronic inflammation and metabolic alterations associated with increased body weight. In addition to cytokines released from inflammatory cells, cytokines produced by adipocytes (so called adipokines) have been implicated in the increased risk of cardiovascular disease associated with obesity. Among others, obesity is associated with elevated circulating levels of leptin^2,3^, a prototype adipokine involved in the regulation of body weight and energy expenditure^4^. Leptin receptors (LepRs) expressed on neuronal cells are crucial for the regulation of food intake and energy expenditure^4^. LepRs are also present on vascular endothelial cells forming the blood–brain barrier^5^. Previous studies in mice expressing membrane-bound truncated, LepRs on endothelial cells showed altered leptin brain tissue uptake and partial resistance to diet-induced obesity^6–8^.

Leptin has functions beyond its activities as a satiety factor. In this regard, endothelial cells lining peripheral blood vessels also express LepRs^9^. Experimental as well as clinical studies have implicated leptin in the pathophysiology of endothelial dysfunction^3^ and other cardiovascular pathologies^10,11^. We previously reported that mice lacking LepRs on endothelial cells exhibit a vascular phenotype mimicking that of leptin-resistant, obese mice^12^. In those mice, we observed a more pronounced visceral adiposity in response to high-fat diet feeding. This and other recent experimental evidence suggest that endothelial cells not only to respond to obesity, but also may play an active role in whole body energy homeostasis and the development of obesity^13–15^. However, few studies have examined this concept, and the mechanisms and mediators are only beginning to emerge^16^. In this study, we examined the hypothesis that genetic deletion of LepR in endothelial cells alters the metabolic phenotype of mice and actively contributes to the development of high-fat diet (HFD) diet-induced obesity.

## Results

### Generation of mice with inducible deletion of LepR in endothelial cells

**Figure 1:**
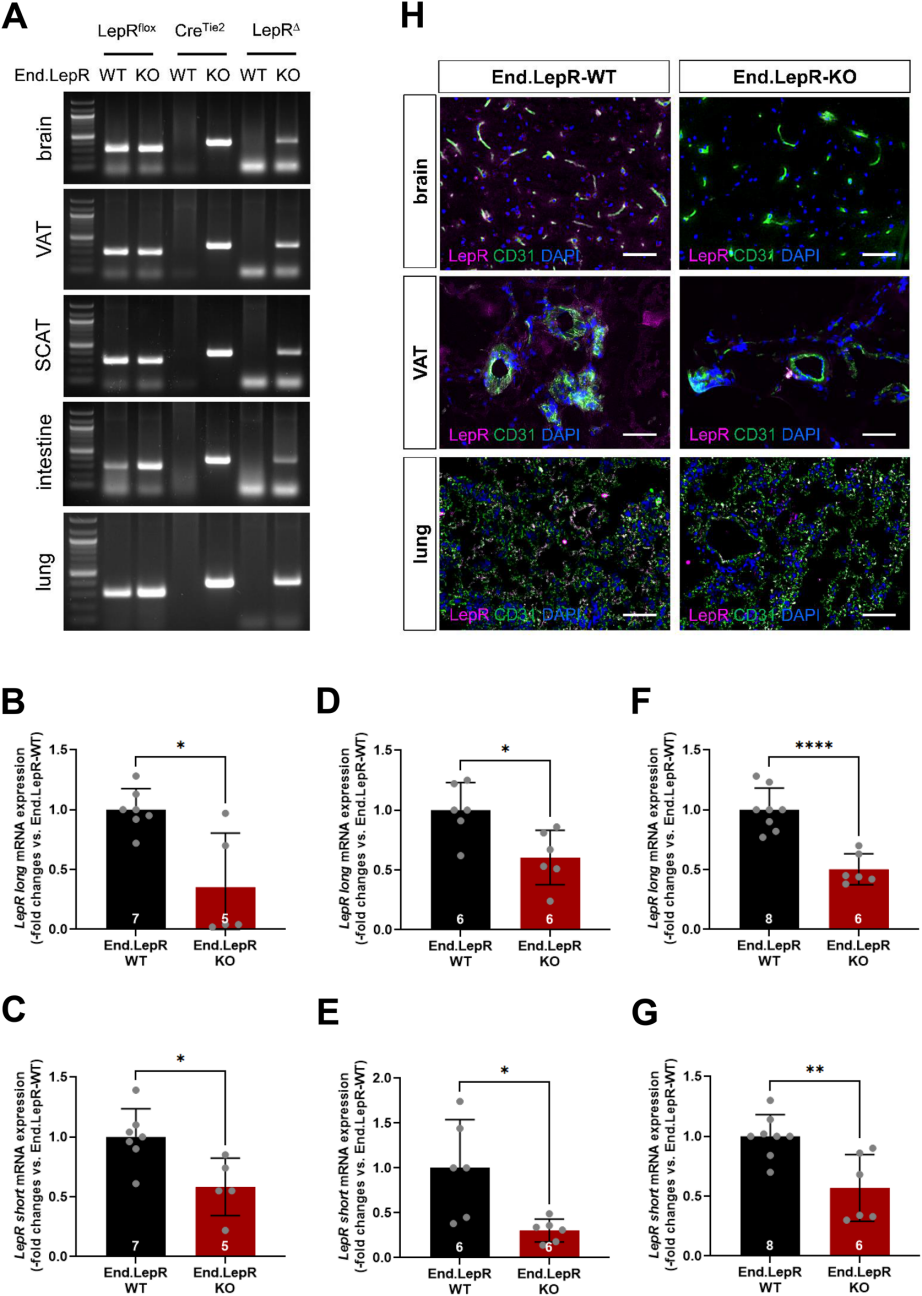
Generation of mice with inducible genetic deletion of LepR in endothelial cells. (**A**) Tamoxifen-induced, Cre recombinase-mediated gene excision of leptin receptors (∆LepR) in brain, visceral adipose tissue (VAT), subcutaneous adipose tissue (SCAT), small intestine and lung tissue biopsies. The full-length images of the gels from which the areas of interest showing bands were cropped are shown in the Supplementary information file. Quantitative real-time PCR analysis of LepR, long (**B**,**D**,**F**) and short (**C**,**E**,**G**) isoform, mRNA levels in endothelial cells isolated from brain (**B**,**C**; n = 5–7 mice per group), VAT (**D**,**E**; n = 6 mice per group) or lung (**F**,**G**; n = 6–8 mice per group) of End.LepR-WT and End.LepR-KO mice. Data were analyzed using Student’s *t* test (**C**–**G**) or Mann–Whitney test (**B**), depending on the presence of normal distribution. *p < 0.05, **p < 0.01 and ****p < 0.001. (**H**) Representative fluorescence microscopy images of LepR expression (magenta) on CD31-immunopositive endothelial cells (green) in brain, VAT and lungs of End.LepR-WT and End.LepR-KO mice. Cell nuclei were visualized using DAPI (blue). Size bars represent 20 µm.

Mice with inducible deletion of LepRs in endothelial cells (End.LepR knockout, KO) were generated previously and examined regarding their vascular^12^ or cardiac^17^ response to injury. PCR analysis of genomic DNA demonstrated inducible Tie2.ER^T2^-Cre-mediated deletion of the floxed LepR gene (visible as LepR delta or Δ) in organs involved in the control of body weight, such as brain or visceral (VAT) and subcutaneous (SCAT) adipose tissue, as well as in other organs, such as small intestine and lung (Fig. 1A). Quantitative real time PCR analysis confirmed significantly reduced mRNA transcript levels of both, the long (signaling) and the short, LepR isoforms in primary endothelial cells isolated from the brain (Fig. 1B,C), VAT (Fig. 1D,E) and lung (Fig. 1F,G) of End.LepR-KO mice, and low LepR immunosignals in CD31-positive endothelial cells were confirmed using immunofluorescence microscopy (Fig. 1H).

### Mice lacking endothelial leptin receptors develop more pronounced obesity in response to high-fat diet

**Figure 2:**
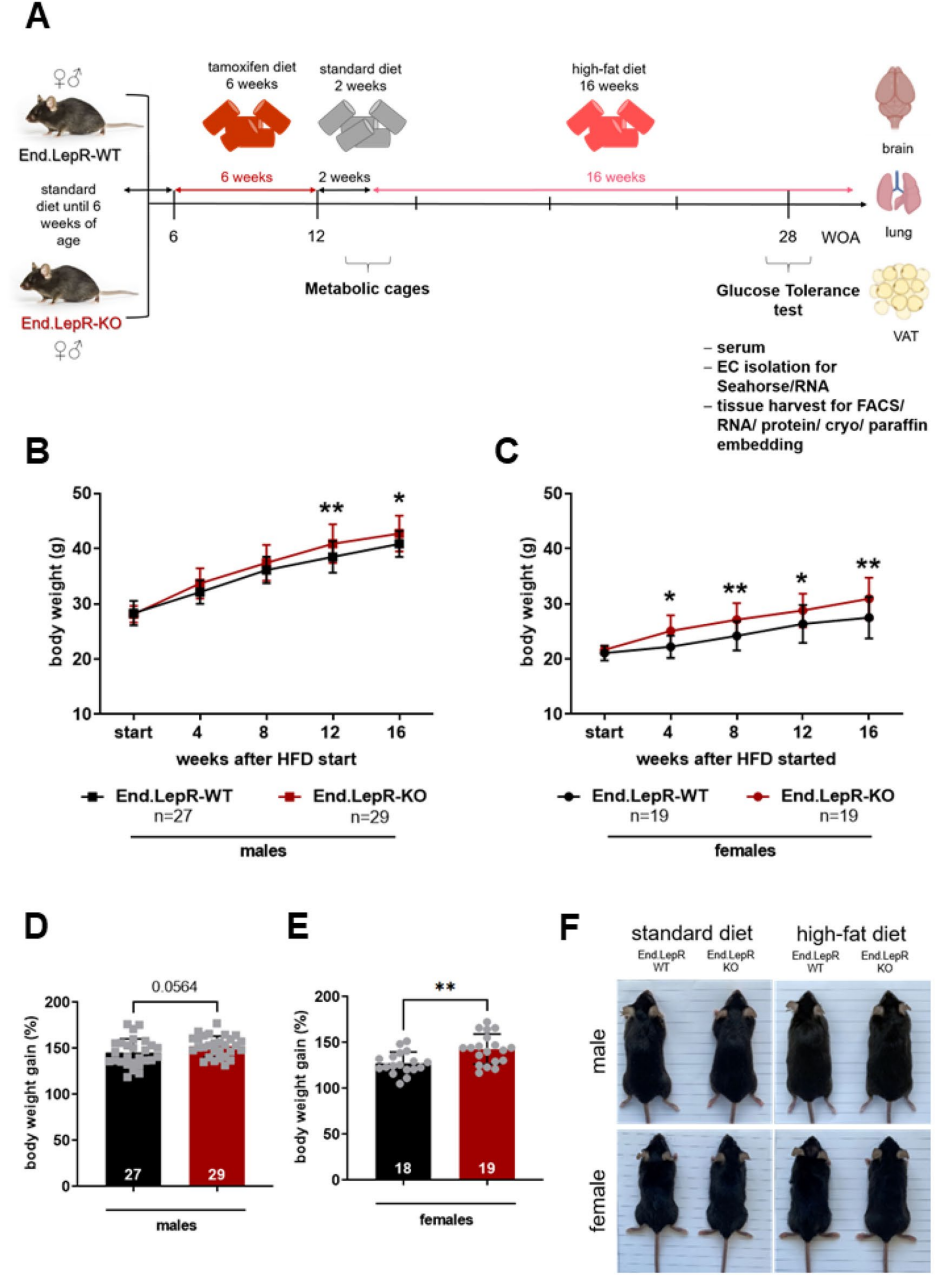
Body weight changes in response to high-fat diet. (**A**) Experimental work flow. Body weight progression curves of male (**B**) and female (**C**) End.LepR-WT and End.LepR-KO mice at the start and after feeding high-fat diet (HFD) for 4, 8, 12 and 16 weeks. The number of mice examined per group is shown in the graphs. *p < 0.05 and **p < 0.01, as determined using Two-Way ANOVA, Sidak’s multiple comparison test. Body weight gain after 16 weeks of HFD, expressed as percentage of initial body weight (set at 100%), in male (**D**) and female (**E**) End.LepR-WT and End.LepR-KO mice. **p < 0.01, as determined using Student’s *t* test. (**F**) Representative photographs of male and female End.LepR-WT and End.LepR-KO mice fed standard laboratory diet or high-fat diet for 16 weeks.

The experimental work flow to examine the role of endothelial leptin receptors for diet-induced obesity in male and female End.LepR-WT and End.LepR-KO mice is shown in Fig. 2A. In mice fed SD, mean body weights did not significantly differ between End.LepR-WT and End.LepR-KO mice (males: 28.4 ± 0.4 vs. 28.1 ± 0.3 g, P = 0.5623; females: 21.1 ± 0.3 vs. 21.6 ± 0.2 g, P = 0.1210). Although all mice developed obesity in response to HFD, mean body weight changes and the percentage of weight gain over 16 weeks were significantly higher in End.LepR-KO mice compared to age-matched End.LepR-WT controls, in both male (Fig. 2B,D) and female (Fig. 2C,E) mice. The observed differences were moderate; representative images of End.LepR-WT and End.LepR-KO littermate mice fed either SD or HFD for 16 weeks are shown in Fig. 2F.

### More pronounced visceral adiposity and hyperleptinemia in female End.LepR-KO mice

**Figure 3:**
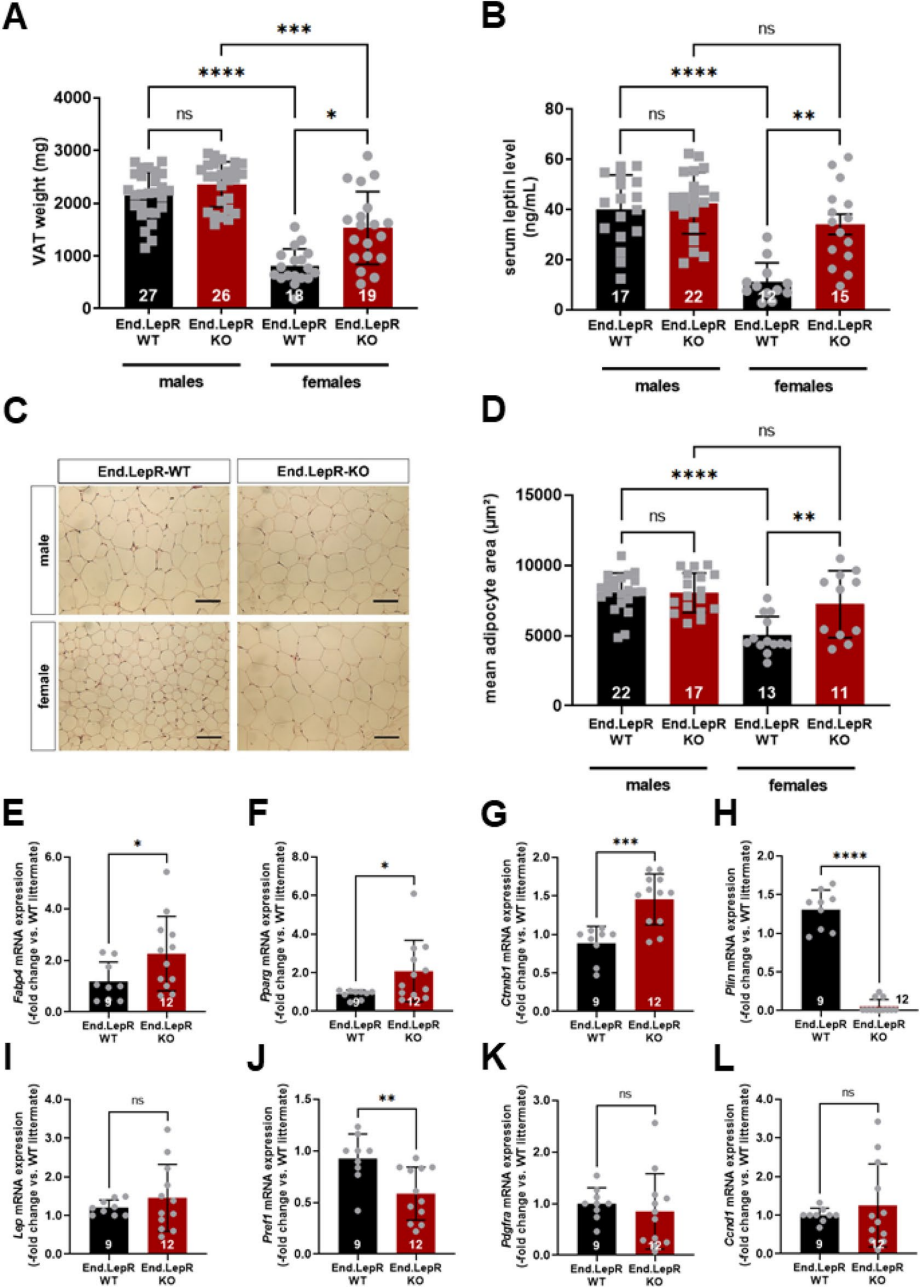
Visceral adiposity, hyperleptinemia and adipogenic gene expression. Visceral adipose tissue (VAT) weights (**A**) and serum leptin levels (**B**) in male and female End.LepR-WT and End.LepR-KO mice fed high-fat diet (HFD) for 16 weeks. Data were compared using Kruskal–Wallis test, Dunn’s multiple comparisons test. *p < 0.05, ***p < 0.001 and ****p < 0.0001. ns, non-significant. Two-Way ANOVA analysis showed significant sex-by-deletion interaction (p = 0.0152 in A, p = 0.0024 in B). (**C**) Representative microscopic images of H&E-stained, paraffin-embedded VAT tissue sections from End.LepR-WT and End.LepR-KO mice. Scale bars represent 200 µm. The results of the morphometric analysis of the mean adipocyte area are shown in (**D**). Data were compared using One-Way ANOVA, Sidak’s multiple comparisons test. **p < 0.01 and ****p < 0.0001. Two-Way ANOVA analysis showed significant sex-by-deletion interaction (p = 0.0117). Relative mRNA expression of Fatty acid-binding protein-4 (*Fabp4*; **E**), Peroxisome proliferator-activated receptor gamma (*Pparg*; **F**), Catenin beta-1 (*Ctnnb1*; **G**), Perilipin-1 (*Plin*; **H**), Leptin (*Lep*; **I**), Preadipocyte factor 1 (*Pref-1*; **J**), Platelet-derived growth factor alpha (*Pdgfra*; **K**) and Cyclin D1 (*Ccnd1*; **L**) in VAT of female End.LepR-KO mice (n = 12) after 16 weeks of HFD. Data are reported as gene expression relative to female End.LepR-WT littermate mice (n = 9) using the ΔΔCt method after normalization to the expression of the reference gene (RPLO). Data were analyzed using Student’s *t* test (**F**,**H**) or Mann–Whitney test (**E**,**G**). *p < 0.05, **p < 0.01, ***p < 0.001 and ****p < 0.0001.

After being fed HFD for 16 weeks, mice were killed. At this time point, visceral adipose tissue (VAT) weights were significantly different only in female End.LepR-KO mice, whereas they were higher in males compared to females and did not significantly differ between End.LepR-WT and End.LepR-KO mice (Fig. 3A). In line with these findings, total circulating levels of leptin, an indicator of peripheral adipose tissue mass^18^, were significantly increased only in female End.LepR-KO mice compared to End.LepR-WT controls and did not differ in males (Fig. 3B). Plasma levels of soluble leptin receptor (sLepR), the main binding protein and inhibitor of leptin^19,20^, did not differ between male and female End.LepR-WT and End.LepR-KO mice (not shown). Histological analysis of H&E stained cross-sections through VAT confirmed an increased mean area of individual adipocytes in female End.LepR-KO mice and a higher, but similar mean adipocyte area in males (Fig. 3C,D). More pronounced adipocyte hypertrophy in the absence of endothelial LepR was confirmed on the molecular level: QPCR analysis of total VAT homogenates from female mice showed that adipocyte mRNA levels of Fatty acid-binding protein-4 (*Fabp4*) (Fig. 3E), Peroxisome proliferator-activated receptor-gamma (*Pparg*) (Fig. 3F) and Catenin beta-1 (*Ctnnb1*) (Fig. 3G), involved in lipid transport or lipogenesis^21^, also were significantly increased, whereas mRNA levels of perilipin (*Plin*), controlling lipolysis, were significantly reduced (Fig. 3H) in End.LepR-KO mice. On the other hand, mRNA levels of leptin did not significantly differ (Fig. 3I). Of note, *Fabp4* is also expressed on microvascular endothelial cells involved in fatty acid transport within adipose tissue^22^. In this regard, *Fabp4* mRNA levels did not significantly differ between endothelial cells isolated from VAT of End.LepR-WT and -KO mice (P = 0.616; n = 5 biological replicates per group; not shown). In line with enhanced adipocyte differentiation, mRNA levels of preadipocyte factor 1 (*Pref1*, also known als *Dlk1*) were significantly reduced in End.LepR-KO mice (Fig. 3J), whereas mRNA levels of platelet-derived growth factor-alpha (*Pdgfra*; Fig. 3K), a marker of fibroblasts and adipocyte precursor cells^23^, or markers of cell proliferation, such as Cyclin D1 (*Ccnd1*; Fig. 3L) or Proliferating cell nuclear antigen (*Pcna*; not shown) did not significantly differ.

### Mice with genetic endothelial LepR deletion exhibit more pronounced VAT inflammation

**Figure 4:**
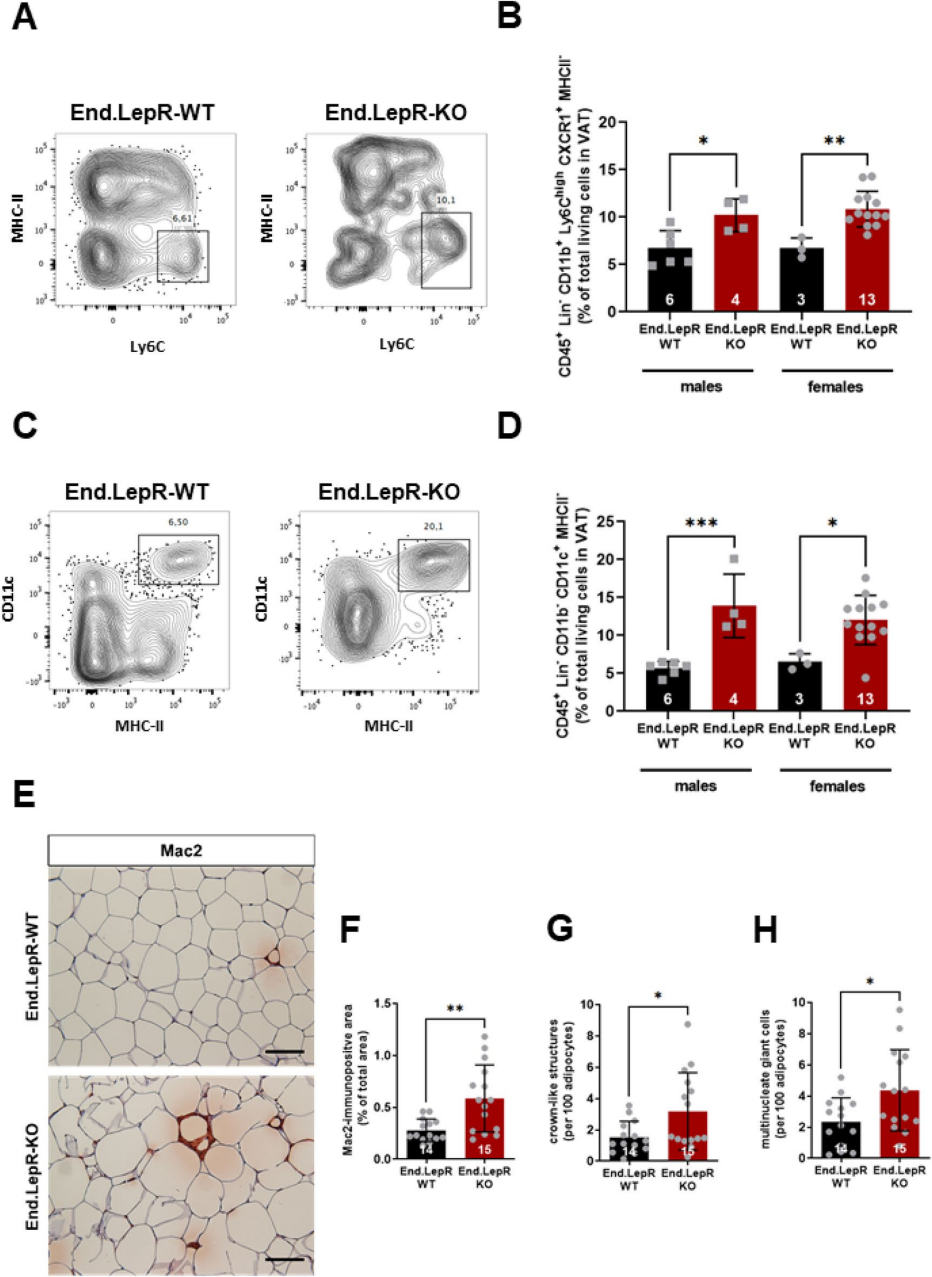
Visceral adipose tissue inflammation. Immune cell subsets were analyzed in visceral adipose tissue (VAT) homogenates of End.LepR-WT and End.LepR-KO mice fed high-fat diet (HFD) for 16 weeks using flow cytometry. Representative dot plots after analysis of MHC-II and Ly6C are shown in (**A**), of CD11c and MHC11 in (**C**), the results of the quantitative analysis of CD45^+^ Lin^−^ CD11b^+^ Ly6C^high^ CXCR1^+^ MHCII^−^ cells in (**B**) and of CD45^+^ Lin^−^ CD11b^−^ CD11c^+^ MHCII^−^ in (**D**), respectively. Data are expressed as % of total living cells in VAT and were compared using One-Way ANOVA, Sidak’s multiple comparisons test. *p < 0.05, **p < 0.01 and ***p < 0.005. (**E**) Representative images after immunohistochemical analysis of macrophages in VAT of End.LepR-WT and End.LepR-KO mice fed HFD for 16 weeks. Scale bars represent 100 μm. The results of the quantitative analysis of the Mac2-immunopositive area are shown in (**F**), the number of crown-like structures (CLS) or nucleated giant cells per 100 adipocytes are shown in (**G**) and (**H**), respectively. Data were analyzed using Student’s *t* test. *p < 0.05 and **p < 0.01.

Regarding differences in the degree of VAT inflammation associated with obesity, flow cytometry revealed significantly higher number of CD45^+^ Lin^−^ CD11b^+^ Ly6C^high^ CXCR1^+^ MHCII^−^ inflammatory monocytes (Fig. 4A,B) and CD45^+^ Lin^−^ CD11b^−^ CD11c^+^ MHCII^+^ monocytes (Fig. 4C,D) in VAT from End.LepR-KO mice compared to End.LepR-WT controls. Immunohistochemistry confirmed higher numbers of Mac2-positive macrophages (Fig. 4E,F), organized as crown-like structures (CLS; Fig. 4G) or fused to multinucleated giant cells (MGS; Fig. 4H)^24^. The total number of CD31-immunopositive endothelial cells in VAT did not differ between End.LepR-WT and End.LepR-KO mice (Suppl. Fig. 1A,B). As a result of the larger mean adipocyte area resulting in a significantly reduced number of adipocytes per microscope field (P = 0.0077 vs. End.LepR-WT in females; P = 0.8359 vs. End.LepR-WT in males; data not shown), the relative number of CD31-immunopositive cells per adipocyte was significantly increased in female End.LepR-KO mice (Suppl. Fig. 1C). Messenger RNA levels of markers of endothelial cell activation did not significantly differ between endothelial cells from End.LepR-WT and End.LepR-KO mice (Suppl. Fig. 2A–C).

### No overt metabolic alterations in mice lacking endothelial LepR

Obesity and chronic (adipose tissue) inflammation are often complicated by metabolic alterations, such as insulin resistance and diabetes mellitus. In this regard, analysis of serum glucose and insulin levels in End.LepR-WT and End.LepR-KO mice fasted for six hours did not reveal differences in serum glucose (Suppl. Fig. 3A) or serum insulin (Suppl. Fig. 3B) levels. Glucose tolerance tests confirmed no significant differences in mice fed SD (Suppl. Fig. 3C) or HFD for 16 weeks (Suppl. Fig. 3D), although a trend towards faster glucose removal was observed in the latter. Also, mean liver weights did not differ between End.LepR-WT and -KO mice (Suppl. Fig. 4A), and the histological analysis of cryo-embedded cross-sections following staining with Oil red O revealed similar amounts of stored neutral lipids (Suppl. Fig. 4B,C).

### Higher total food intake and whole-body energy homeostasis in mice lacking endothelial leptin receptors

**Figure 5:**
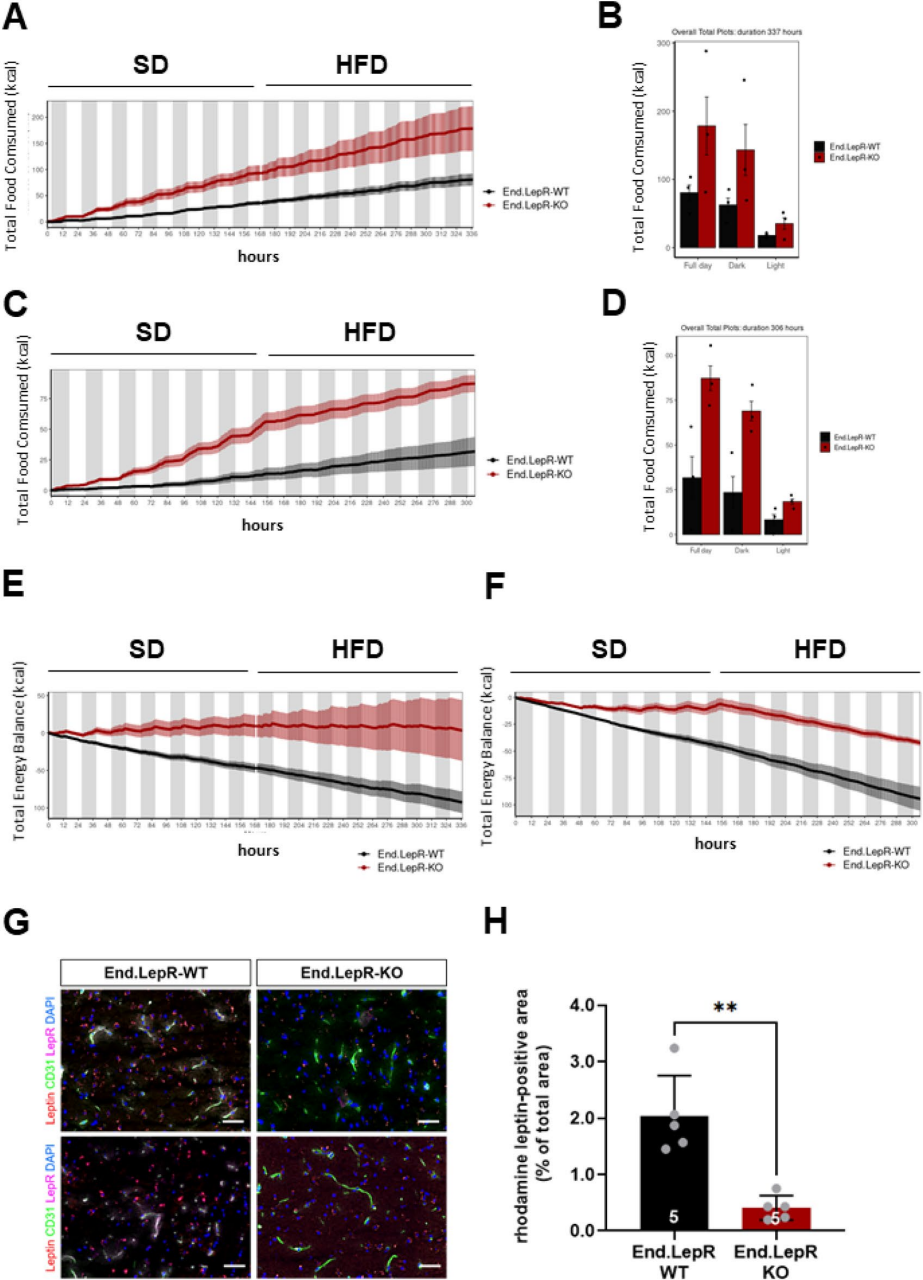
Total food consumption, energy balance and transport of exogenous leptin into the brain. Total food intake time plot of male (**A**) and female (**C**) End.LepR-WT and End.LepR-KO mice (n = 3 per group) recorded in metabolic cages in 12 h day–night repeated cycles. Mice were fed standard laboratory diet (SD) for 7 days followed by a switch to high-fat diet (HFD) for additional 7 days. Summary of total food consumed over a duration of 14 days for male (**B**) and female (**D**) End.LepR-WT and End.LepR-KO mice. Time plot of total energy balance before and after diet switch from SD to HFD in male (**E**) and female (**F**) End.LepR-WT and End.LepR-KO mice. (**G**) Representative fluorescence microscopy images of cryo-embedded brain sections of End.LepR-WT and End.LepR-KO mice, i.p. injected with rhodamine-labeled leptin (red) and i.c. injected with FITC-labeled lectin to visualize endothelial cells (green) immunostained for LepR (magenta). DAPI-positive cell nuclei are blue. (**H**) Summary of the quantitative analysis of the rhodamine leptin-positive area per total area of 40 × microscopic fields. **p = 0.01, as determined using Student’s *t* test.

To examine whether differences in daily food consumption, energy expenditure or physical activity have contributed to the more pronounced obesity in End.LepR-KO mice, indirect calorimetry was employed. Individual male and female littermate mice were observed for 1 week on SD followed by 1 week observation on HFD (n = 3 independent experiments; n = 12 biological repeats). End.LepR-KO mice exhibited a higher total food consumption (Fig. 5A–D) and total energy balance (Fig. 5E,F). On the other hand, daily physical activity, measured as pedestrian locomotion (not shown), locomotor activity (Suppl. Fig. 5A,B) or total distance in cage locomotion (Suppl. Fig. 5C,D), did not significantly differ. Oxygen consumption (VO_2_; Suppl. Fig. 6A,B), carbon dioxide production (VCO_2_; Suppl. Fig. 6C,D) and respiratory exchange rates (Suppl. Fig. 6E,F) also were similar, indicating unaltered oxidation of energy substrates in End.LepR-KO mice.

### Deletion of endothelial leptin receptors impairs leptin’s transport across the blood–brain barrier

The sensing of circulating levels of the satiety hormone leptin requires its binding to leptin receptors expressed on cells of the blood–brain barrier followed by transcytosis^25–27^. Fluorescence microscopy analysis demonstrated the binding of exogenous, rhodamine-labeled recombinant leptin to endothelial cells lining the median eminence and throughout the brain in End.LepR-WT mice, whereas low signals of exogenously administered leptin were detected in the brain of End.LepR-KO mice. Representative examples are shown in Fig. 5G, the results of the quantitative analysis in Fig. 5H. These findings suggested that the more pronounced obesity and higher food intake observed in mice lacking endothelial LepR has occurred as consequence of an impaired transcytosis of the satiety factor across the blood–brain barrier. Analysis of VAT tissue sections revealed similar findings, that is significantly reduced rhodamine-labeled leptin in End.LepR-KO mice (Suppl. Fig. 7A,B).

### Absence of leptin receptors alters endothelial cell metabolism in an organ-specific manner

**Figure 6:**
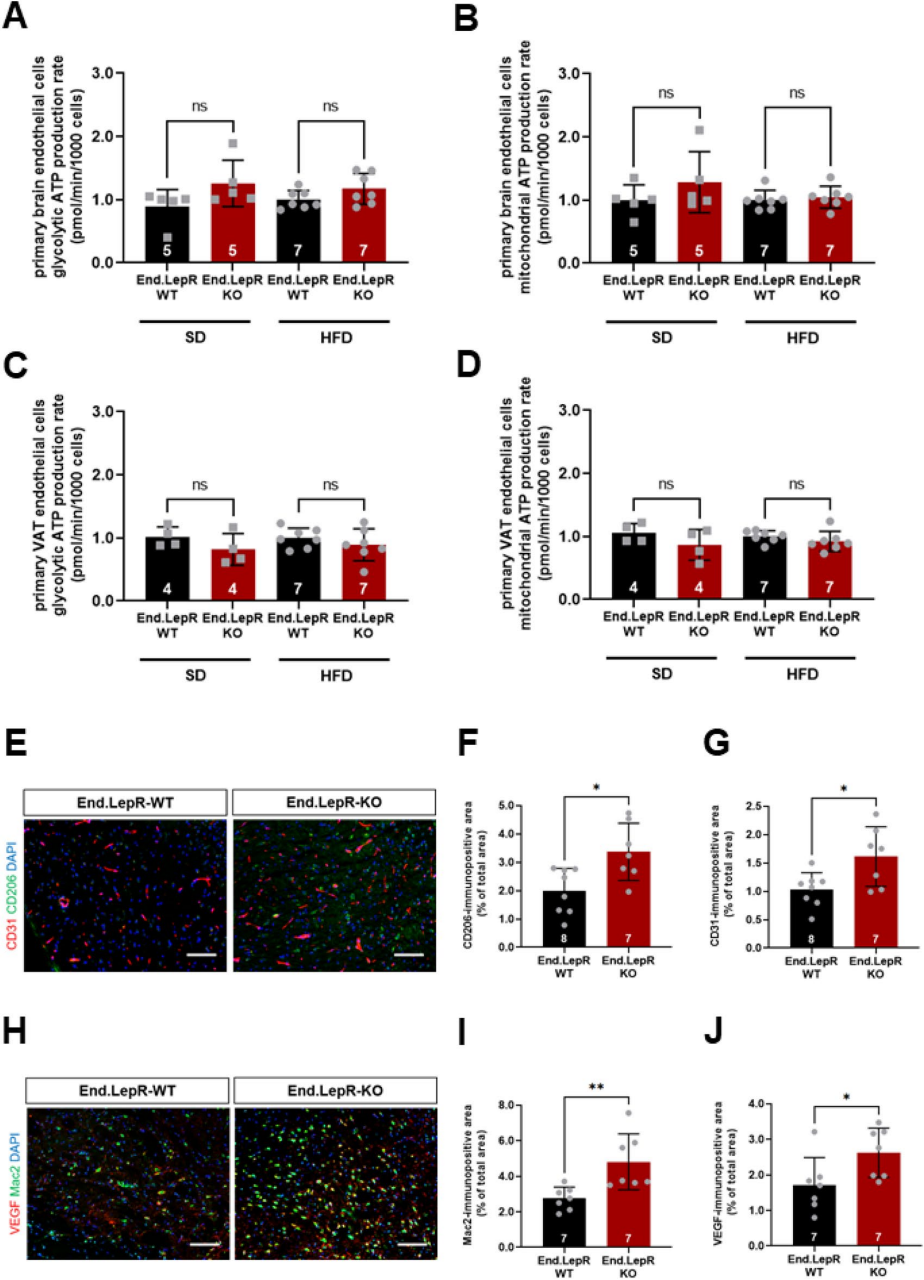
Brain inflammation and energetic profiles of primary brain and VAT endothelial cells. Summary of findings after analysis of glycolysis and mitochondrial respiration in living primary endothelial cells isolated from brain (A,B) or visceral adipose tissue (VAT; **C**,**D**) of female End.LepR-WT (n = 4–5) and End.LepR-KO (n = 7) mice fed standard laboratory diet (SD) or high-fat diet (HFD) for 16 weeks. Analyses were performed using the Seahorse XF96e Extracellular Flux analyzer. Results were normalized to the number of cells and are expressed as -fold change vs. End.LepR-WT mice. Data were analyzed using One-Way ANOVA, Sidak’s multiple comparisons test. Ns, non-significant. Representative immunofluorescence images of cryo-embedded brain sections from End.LepR-WT (n = 7–8) and End.LepR-KO (n = 7) mice, fed high-fat diet for 16 weeks, after staining with antibodies against CD206 (green) and CD31 (red) (**E**) or against Mac2 (green) and VEGF (red) (**H**). Size bars represent 20 µm. Results after quantification of the area immunopositive for CD206 (**F**), CD31 (**G**), Mac2 (**I**) or VEGF (**J**), expressed per total area of 40 × microscopic fields. Data were analyzed using Student’s *t* test. *p < 0.05 and **p < 0.01.

To further study the role of LepR on endothelial cells for the control of body weight, beyond the regulation of leptin transcytosis, primary endothelial cells were isolated from brain and VAT of End.LepR-KO and End.LepR-WT mice and their bioenergetic profiles examined in *real time*. However, the extracellular acidification rate (ECAR; an indicator of glycolysis) and the oxygen consumption rate (OCR; an indicator of mitochondrial respiration) did not significantly differ between endothelial cells isolated from the brain of End.LepR-WT and -KO mice, neither in mice fed SD nor in those fed HFD (Fig. 6A,B; raw data are shown in Suppl. Fig. 8A,B). ECAR and OCR also were similar in endothelial cells isolated from VAT of End.LepR-KO mice compared to End.LepR-WT controls (Fig. 6C,D; raw data are shown in Suppl. Fig. 8C,D).

Previous studies suggested a role of inflammatory cell derived cytokines in the maintenance of endothelial cell metabolism in obesity^28^. Immunofluorescence microscopy analysis of brain sections showed that HFD feeding was associated with high numbers of inflammatory cells, similar to VAT. Specifically, the number of CD206-immunopositive cells in the vicinity of CD31-immunopositive endothelial cells (Fig. 6E–G) as well as the number of Mac2-immunopositive macrophages expressing VEGF (Fig. 6H–J) was higher in End.LepR-KO mice compared to End.LepR-WT mice.

**Figure 7:**
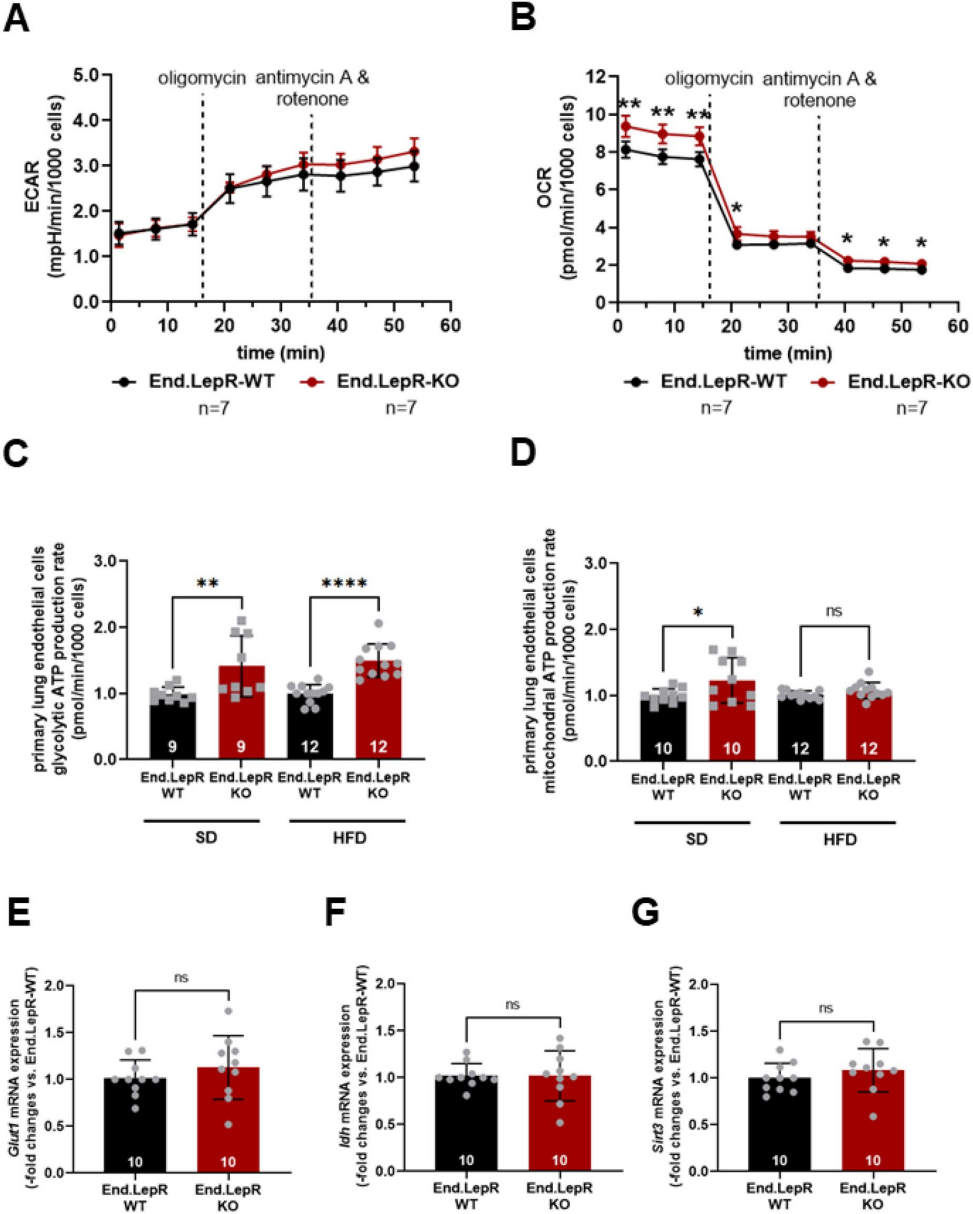
Energy metabolism and glycolytic enzyme or mitochondrial marker expression in primary lung endothelial cells. Representative examples of the extracellular acidification rate (ECAR; **A**) and the oxygen consumption rate (OCR; **B**) in live primary endothelial cells isolated from female End.LepR-WT (n = 7) and End.LepR-KO (n = 7) mice fed standard diet. Data were analyzed using Two-Way ANOVA, Sidak’s multiple comparison tests. *p < 0.05 and **p < 0.01 vs. End.LepR-WT mice at the same time point. Non-significant differences are not shown. The summary of findings from n = 4 independent experiments measuring the ECAR (**C**) or OCR (**D**) to determine glycolysis or mitochondrial respiration in End.LepR-WT and End.LepR-KO mice fed standard diet (SD) or high-fat diet (HFD). Results were normalized to the number of cells and are expressed as -fold change vs. End.LepR-WT mice. Data were compared using One-Way ANOVA, Sidak’s multiple comparison tests. *p < 0.05, **p < 0.01 and ****p < 0.0001 vs. End.LepR-WT mice. Quantitative real-time PCR analysis of *Glut1* (**E**), *Idh* (**F**) and *Sirt3* (**G**) in primary lung endothelial cells from female End.LepR-WT and End.LepR-KO mice (n = 10 per group). Data were compared using Student’s *t* test. *Ns* non-significant.

In contrast to findings in brain and VAT, the glycolytic and mitochondrial ATP production rates were significantly increased in endothelial cells isolated from the lung of End.LepR-KO mice, independent of the diet (Fig. 7A–D). However, qPCR analysis of the mRNA levels of glucose transporters (i.e. *Glut1*; Fig. 7E) or glycolytic enzymes (i.e. *Hk2*, *Pfkb3*; not shown), as well as mitochondrial markers (i.e. *Idh*; Fig. 7F), regulators of mitochondrial function (i.e. *Sirt3*; Fig. 7G) or activators of mitochondrial biogenesis (i.e. *Pcg1α*; not shown) did not significantly differ between primary ECs isolated from lungs of End.LepR-WT and -KO mice, suggesting alternative pathways of metabolic activation.

## Discussion

The adipokine leptin controls body weight and energy expenditure, primarily by acting on LepRs on neuronal cells^29^. Leptin receptors are expressed on many different cell types throughout the body^30^. Here, we show that leptin receptors on endothelial cells participate in the regulation of food intake and body weight and identify reduced transport of leptin across the blood–brain barrier as one potential mechanism. Elevated circulating leptin levels despite increased body weight confirmed the presence of leptin resistance in mice lacking endothelial leptin receptors. Other, indirect consequences of the increased body weight were also more pronounced, such as VAT inflammation and adipogenic gene expression, whereas serum glucose or insulin, whole body metabolism or physical activity levels were not altered. The observed changes were moderate and measurable only in mice on obesogenic diet, suggesting that endothelial LepRs are not causally involved in the development of obesity, but rather play a modifying role.

The regulation of body weight and energy homeostasis is a complex process, which is controlled in the central nervous system and at the level of peripheral organs, including adipose tissue, skeletal muscle, and liver^31^. A role of leptin and its receptors in the control of food intake and energy expenditure was suggested several years ago by findings of severe obesity in leptin-deficient *ob/ob* mice or in *db/db* mice expressing mutated, non-functional LepRs on all cells throughout the body. Underlining the primary role of leptin receptors on neuronal cells in body weight homeostasis, selective genetic deletion of LepR in all neurons (using *synapsin I-*Cre)^32^ or defined neuronal cell populations, such as those expressing agouti-related peptide^33^, but not in hepatocytes^32^, caused severe obesity and diabetes, and the phenotype of *db/db* mice could be rescued by neuronal re-expression of leptin receptors^34,35^. Although the presence of LepR on neuronal cells was shown to be ‘both required and sufficient’^36^, a role for leptin receptors on non-neuronal cells in the control of body weight has also been reported^37^. Previous genetic and pharmacological ablation studies in mice identified leptin signaling in astrocytes^38,39^ or oligodendrocyte precursors/glial cells^40^ to play a role in the maintenance of a normal energy homeostasis. Other LepR expressing cells within the brain, such as perivascular^41,42^ or endothelial cells^5,43^, also have been implicated in the central nervous system control of body weight by acting as mediators of leptin uptake. Genetic deletion of LepR using *Slco1c1* (Solute Carrier Organic Anion Transporter Family Member 1C1), a transmembrane receptor expressed on brain capillary endothelial cells^44^, as Cre driver revealed that the transport of radioactive-labeled leptin into the hypothalamus was reduced by about 40%^36^. Although no response to exogenous leptin was observed, body weight gain monitored over 3 months was affected only in mice fed HFD^36^, similar to our findings. Slightly increased plasma leptin levels and body weight were also observed in HFD-fed mice with global deletion of short LepR isoforms^45^. Although the endothelial expression of both LepR isoforms was reduced in our study, these findings suggest that absence of the short LepR isoform, that is the isoform broadly expressed on peripheral cells^46^, may have been sufficient^47^.

Leptin receptors are expressed on endothelial cells throughout the body^48^. To examine their role, previous studies used mice constitutively expressing membrane-bound, truncated LepRs in Tie2-expressing cells^6,8^, that is endothelial cells but also hematopoietic cells. These so-called ELKO mice were partially protected from obesity and exhibited a moderately improved metabolic phenotype in response to HFD despite elevated blood leptin levels^8^. In contrast to these previous reports, mice in which the first exon was flanked by loxP sequences to completely delete LepR following Cre recombination^32^ were examined in the present study, and Cre recombinase activity was induced in adult mice to selectively target Tie2-expressing endothelial, and not hematopoietic cells^49^. Accordingly, HFD was also started later, and not at age 6 weeks^8^, thus avoiding the weight gain normally occurring during growth.

Obesity in humans occurs as the result of an imbalance between energy intake and expenditure, possibly as a consequence of impaired leptin signaling in the presence of elevated circulating leptin levels. Indirect calorimetry demonstrated significant differences in food intake and total energy balance in End.LepR-KO mice fed HFD, in line with ‘perceived’ low leptin levels following endothelial LepR deletion and an impaired transport of leptin across the blood–brain barrier, one of the mechanisms underlying the development of leptin resistance in obesity^50^. Of note, leptin transport across the blood–brain barrier was not found to be altered in mice expressing a truncated form of LepR in Tie2-expressing cells or astrocytes^7^. Some studies suggested the leptin transport across the blood–brain barrier to be intact in obese mice^51^ or that leptin may reach the brain via direct transport through circumventricular organs^52^. Tanycytes, a specialized glial cell type lining the floor of the third ventricle, have been suggested to mediate the transport of peripherally administered leptin into the brain^53^, and tanycyte-specific deletion of LepRs caused hyperleptinemia and increased body weight gain^54^, although other laboratories found leptin-induced changes in hypothalamic STAT3 signaling not to depend on tanycytes^55^. Of note, the transport of leptin across the blood–brain barrier does not only depend on LepRs, but also on its interaction with other receptors, such as EGFR, or the binding of leptin to other receptors, such as megalin/LRP2^56,57^. Although the affinity of leptin to bind to those receptors has been shown to be much lower than for LepR^58^, these mechanisms may become relevant in states of hyperleptinemia.

In line with the development of (functional) leptin resistance in End.LepR-KO mice, obesity was associated with elevated leptin levels. Although leptin mRNA expression levels did not differ between in VAT of End.LepR-WT and End.LepR-KO mice, corroborating findings in ELKO mice^6^, an increased number of leptin-producing fat cells may have contributed to this observation. In addition to being a consequence of the increased adipose tissue mass, elevated leptin levels may have developed as a consequence of the reduced endothelial LepR uptake into the brain or other organs^6^. Regarding our observation of increased visceral adiposity and hyperleptinemia primarily in female mice, we attribute them the fact that HFD was started relatively late in life, and that differences in these parameters may not have been detectable in the already heavier, male mice. Previous in situ hybridization studies revealed no differences in LepR expression in the arcuate nucleus, ventromedial nucleus, thalamus and piriform cortex between male and female rats, although LepR gene expression decreased in response to estradiol administration and increased following ovarectomy^59^.

With respect to the role of leptin receptors on endothelial cells beyond the control of leptin transport to neuronal centers of body weight control, we have shown that inducible genetic deletion of endothelial leptin receptors in mice or siRNA-mediated downregulation of leptin receptors in human endothelial cells leads to cardiac endothelial cell autophagy, similar to mTOR inhibition or cellular starvation^17^. In the present study, metabolic changes in endothelial cells were observed only in those isolated from peripheral organs not known to be involved in the control of body weight, such as the lungs, whereas energy substrate handling was unaltered in those isolated from brain and VAT. Factors produced in inflammatory cells may have preserved the endothelial metabolic functions and acted as protective mechanism, as recently shown for VEGF-producing perivascular macrophages in the obese mouse brain^28^. Single-cell sequencing analysis recently showed organ-specific differences in the susceptibility to obesity^60^, which also may have played a role, although this could not be examined in the present study. Also, absence of LepR on endothelial cells may have affected adipose metabolism by altering sympathetic fat innervation^61^ or angiocrine mediators^62^.

Taken together, our findings support a role for endothelial LepRs in mediating the transport of leptin into the brain and the neuronal control of food intake, but not whole-body energy metabolism, whereas energy production in endothelial cells was altered in an organ-specific manner.

## Methods

### Experimental animals

The generation of mice with tamoxifen-inducible, Tie2.Cre-ER^T2^-mediated deletion of LepR in endothelial cells was described previously^12,17^. For Cre recombinase activation, mice (6 weeks-of-age) were fed tamoxifen citrate-containing rodent chow (Envigo; TD.130860) for 6 weeks^49^. Genomic DNA from the brain, lung, small intestine, subcutaneous adipose tissue (SCAT) and visceral adipose tissue (VAT) was isolated using Direct PCR lysis reagent (Peqlab) containing 0.2 mg/mL proteinase K (Peqlab). The mouse genotype was determined using the following primers: Tie2.Cre (Tie2Cre1: 5′-CGA GTG ATG AGG TTC GCA AG-3′; Tie2Cre2: 5′-TGA GTG AAC GAA CCT GGT CG-3′); LepR (LepRflox1: 5′-GTC ACC TAG GTT AAT GTA TTC-3′; LepRflox2: 5′-TCT AGC CCT CCA GCA CTG GAC-3′. Tamoxifen-induced deletion of LepR gene was confirmed using the primers: LepRflox1: 5′-GTC ACC TAG GTT AAT GTA TTC-3′ and LepR delta/∆: 5′-GCA ATT CAT ATC AAA ACG CC-3′). Sex-matched littermate Tie2.ER^T2^-WT LepR^flox/flox^ mice, fed tamoxifen-containing rodent diet, were used as controls throughout the entire study.

### High-fat diet-induced obesity model

Following tamoxifen diet, littermate male and female mice were fed standard laboratory diet (SD) for mice (V1124-300; ssniff^®^) for 2 weeks and then switched to 45 kcal-% HFD (D12451; Research Diets) ad libitum to induce obesity. Body weight was determined before and every 4 weeks for a total of 16 weeks. All experimental procedures involving animals had been á priori approved by the local animal ethics committee (Translational Animal Research Center, University Medical Center Mainz) and the *Landesuntersuchungsamt Rheinland-Pfalz* (animal permit G16-1-081) and complied with national guidelines for the care and use of laboratory animals. The study is reported in accordance with ARRIVE guidelines.

### Indirect calorimetry

To continuously and simultaneously measure the energy expenditure, physical activity, O_2_ consumption and CO_2_ production as well as food and water intake, a subset of mice on SD was transferred to metabolic cages (Promethion High-Definition Multiplexed Respirometry Cage; Sable Systems™). Each cage was lined with Aspen chips, food hopper and water bottle connected to a food/water intake monitoring system built into the cage lid. Physical activity was monitored in real time using the BXYZ beam break activity monitor system. After an equilibration period of 5 days to standard animal housing conditions, metabolic parameters were recorded over 7 days on SD followed by 45% HFD for additional 7 days. The temperature was kept constant at 22 °C, and lights were set to on and off at 6:00 and 18:00 h respectively, to maintain 12 h light–dark cycles and correlate activity patterns with circadian cycles. Per each experiment (n = 3 experimental replicates), End.LepR-WT and End.LepR-KO (n = 4 mice per experiment) were studied. The *CalR* web tool was used to analyze the indirect calorimetry raw data for generating plots and statistical analysis. Metabolic parameters were normalized to the respective individual body weight of mice recorded at the start of the equilibration stage^63^.

### Glucose tolerance testing

In subsets of littermate mice, glucose tolerance tests were performed after being fed SD or HFD for 14 weeks. Following the removal of food for 6 h (with drinking water ad libitum), as recommended for metabolic studies in mice^64^, mice were injected intraperitoneally with a 20% glucose solution at a dose of 2 g/kg body weight (volume of i.p glucose injection (μL) = 10 × body weight (g)), and (tail vein) blood glucose levels were determined before and 15, 30, 60 and 120 min after the glucose administration using the CONTOUR^®^ NEXT blood glucose monitoring system (Ascensia Diabetes Care Holdings).

### Tissue harvest

After 16 weeks of HFD, mice were deeply anesthetized (using a mixture of ketamine hydrochloride [75 mg/kg body weight] and xylazine hydrochloride [15 mg/kg body weight)] in 0.9% sodium chloride solution), and whole blood was taken by cardiac puncture to measure fasting glucose levels, as above. The rest was allowed to clot for 30 min at room temperature followed by serum preparation. Mice were killed by cervical dislocation under deep anesthesia. Their brain, visceral perigonadal adipose tissue (VAT) and liver were removed, weighed and prepared for subsequent molecular or histological analyses, or endothelial cell isolation. In some mice, rhodamine-labeled leptin (FR-003-13, Phoenix Pharmaceuticals; 5 mg/kg body weight) was injected 45 min before tissue harvest via intraperitoneal (i.p.) injection. Then, fluorescein-labeled Lectin I from Griffonia Simplicifolia (FL-1201, Vector Laboratories) was injected intracardially (i.c.) 15 min before tissue harvest to label functional blood vessel and microglial cells in vivo. Tissues were prepared for cryo-preservation and fluorescence microscopy analysis.

### Determination of serum metabolic parameter

Serum was prepared by centrifugation at 3000 rpm for 10 min and stored at − 80 °C pending analysis. Fasting serum glucose was measured using colorimetric assays (BioAssay Systems). Commercial enzyme-linked immunoassays were employed to determine circulating levels of murine leptin (R&D Systems Inc; detection range: 1.58–5.56 pg/mL), soluble leptin receptor (sLepR; antibodies online, ABIN773812) or insulin (Crystal Chem, #90060; detection range: 0.1–12.8 ng/mL), according to the manufacturer’s instructions.

### Histological and immunohistochemical analysis

For the preparation of paraffin-embedded tissue sections and histology, a portion of VAT was fixed overnight in 4% zinc formalin (Sigma) followed by incubation in 70% ethanol (Carl Roth) and embedded in paraffin (Surgipath^®^ Paraplast; Leica Biosystems). Five µm-thick serial cross sections were deparaffinized and stained with hematoxylin and eosin (H&E) to measure the single adipocyte area. For immunohistochemistry, deparaffinized sections were washed in a series of graded alcohol followed by heat-induced antigen retrieval (in 0.01 M citrate buffer, pH 6.0 for 6 min) prior to blocking with 10% normal serum (abcam). Sections were incubated overnight at 4 °C with primary rat monoclonal antibody against Mac2 (CL8942AP; Cedarlane Laboratories; dilution, 1:400 in antibody diluent (Dako)). For detection, sections were incubated with secondary antibody (dilution: 1:1000; Molecular Probes) followed by avidin–biotin complex (Vector Laboratories) and amino ethyl carbazole substrate (abcam) until color development. Sections were briefly counterstained with Gill’s hematoxylin (Sigma), mounted in ImmuMount (ThermoScientific) and photographed at 20 × magnification (Olympus BX51 microscope). Mac2-immunosignals were quantified as percentage of total area using Image-Pro Plus (version 7.0) software (Media Cybernetics Inc.). For cryo-preservation and the preparation of cryo sections, tissues were immediately embedded in Tissue-Tek O.C.T. compound (Sakura via Science Services GmbH). To analyze lipid accumulation in liver, eight-micrometer thick cryo-sections were fixed in 4% formaldehyde for 15 min, washed three times in 1 × PBS followed by 0.5% Oil red O (Sigma) staining for 1 h at room temperature. The tissues were washed, counterstained with hematoxylin and photographed at 20 × magnification at an inverted light microscope (Motic AE31, Motic).

### Morphometric analysis

The single adipocyte area was determined, as previously described^65^. In brief, 5 µm-thick cross sections through VAT were stained with H&E and photographed at 10 × magnification using an Olympus BX51 microscope. Five representative images were captured from each cross section, and at least 10 adipocytes per image were randomly selected and measured using image analysis software (Image-Pro Plus, version 7.0). Results were averaged per mouse. The Oil red O-positive area on liver sections was determined on 5 images per each animal and the results averaged per mouse.

### Immunofluorescence microscopy analysis

For immunofluorescence analysis, 8 µm-thick cryo-sections were processed, as described below. Sections were defrosted for 5 min at room temperature and washed (2 × 5 min) with 1 × PBS (pH 7.5) followed by fixation in ice-cold acetone (PanReac AppliChem) for 10 min at − 20 °C. Sections were washed and rehydrated with 1 × PBS (3 × 5 min) and permeabilized in 0.05% Triton X-100 (in PBS; Roth) for 10 min at 37 °C. Primary antibody was diluted in antibody diluent (Dako), and sections were incubated overnight at 4 °C. The following primary antibodies were used: anti-Mac2 (CL8942AP; Cedarlane Laboratories), anti-CD206 (ab64693; abcam), anti-VEGF (ABS82, Millipore), anti-LepR (AF498; R&D Systems), and anti-CD31 (sc18916; Santa Cruz Biotechnology). Sections were washed with PBS, followed by incubation with the secondary antibody in 1 × PBS for 2 h at room temperature in the dark. The following secondary antibodies used were: Alexa Fluor™ Plus 647 donkey anti-goat (A32849; Life Technologies), Alexa Fluor™ 488 goat anti-rat (ab150157; abcam), Alexa Fluor™ 555 goat anti-rat (ab150154; abcam), Alexa Fluor™ Plus 488 goat anti-rabbit (A32731, Life Technologies) and Alexa Fluor™ Plus 555 goat anti-rabbit (A32732; Life Technologies). Cell nuclei were visualized using 4′, 6-diamidino-2-phenylindole (DAPI; Sigma). To exclude nonspecific immunostaining, sections were incubated with secondary antibodies alone. Images were acquired using an inverted fluorescence microscope (Keyence; BZ-X810) equipped with a 40 × objective lens and BZ-X800 image software. Immunosignals were quantified using Image-Pro Plus software on at least 5 images per animal, and the results averaged per mouse.

### Mouse endothelial cell isolation and culture

Primary murine endothelial cells (mPECs) were isolated from the brain, lungs and VAT. Primary endothelial cells from the brain and VAT were isolated using Papain Dissociation System (Worthington), according to the protocol provided in the datasheet. Primary murine endothelial cells from lungs of End.LepR-WT and End.LepR-KO mice were isolated using magnetic cell sorting with mouse CD31 microbeads (Miltenyi Biotec), as described previously^10^. Cells were cultured on 0.1% gelatin-coated cell culture plates and maintained in endothelial cell growth medium MV2 (PromoCell) until confluency. Cells were analyzed between passages 0 and 2.

### Endothelial metabolic measurements

To study the total ATP production rate from glycolysis and mitochondrial respiration in live endothelial cells, Seahorse XFp Real-Time ATP Rate Assay was performed using the Seahorse XF96e Extracellular Flux analyzer (Agilent Technologies). One day prior to performing the assay, endothelial cells were plated into gelatin-coated XF96 (V3) polystyrene cell culture plates (Agilent Technologies) and cultured in MV2 medium (PromoCell). After 24 h, medium was changed to XF assay medium (XF RPMI with 1 mM HEPES; Agilent Technologies; #103576-100), supplemented with 1 mM pyruvate, 2 mM l-glutamine and 25 mM glucose (all Sigma-Aldrich), and cells incubated at 37 °C in a SpectraMax i3 (VWR; INCU-Line) used as non-CO_2_ incubator for 45 min, while conduction bright field imaging to document homogenous seeding of cells. After calibration and initialization of the sensor cartridge, three baseline measurements were recorded, prior to the addition of each compound, and three response measurements after sequential injections of the ATP synthase inhibitor oligomycin (1.5 μM) from port A, the complex 1 inhibitor rotenone (0.5 µM) and the complex 3 inhibitor antimycin A (0.5 μM), both from port B. After completion of the assay, cells were stained with BioTracker NIR694 Nuclear Dye (Sigma, #SCT118) and counted for normalization using the SpectraMax MiniMax 300 Imaging Cytometer (Molecular Devices). Cell nuclei were automatically identified using the SoftMaxPro 6.5.1 software by setting size and threshold for object identification in the red channel. Data analysis was performed using Wave 2.6.3.5 Software (Agilent Technologies).

### RNA isolation and quantitative real time PCR analysis

Total RNA was isolated from primary murine endothelial cells using TRI Reagent^®^ (Ambion) solution, as previously described^10^. To isolate total RNA from adipose tissue, freshly harvested murine VAT was cut into small pieces and transferred to a 2 mL RNase/DNase free tube containing 0.5 mL of TRI Reagent and mechanically homogenized (Miccra homogenizer) on ice. The homogenate was centrifuged at 12,000×*g* for 15 min to remove the lipid fraction on the surface of the aqueous phase. The aqueous phase was then transferred to a fresh 1.5 mL tube followed by subsequent RNA isolation steps of phase separation using 100 µL chloroform, mixed vigorously and centrifuged at 12,000×*g* at 4 °C for 15 min. The aqueous layer was transferred to a fresh tube with 500 µL of isopropyl alcohol and mixed to precipitate the RNA. After 20 min of incubation on ice, precipitated RNA was spun down at 12,000×*g* at 4 °C for 10 min. The RNA pellet was washed twice with 75% ethanol and air-dried before being dissolved in RNAse-free water. The concentration and quality of the isolated RNA was checked by spectrometry (Nanodrop; Thermo Scientific). One μg total RNA was reverse transcribed into cDNA using M-MLV reverse transcriptase (Promega) after DNase treatment (Sigma). Quantitative real-time RT-PCR (qRT-PCR) was performed using SYBR^®^ Green (BioRad) and CFXConnect Real-Time PCR Detection System (BioRad). Results were quantified using the ΔΔCt method. Threshold values (C_t_) of genes of interest were first normalized to the reference genes C_t_ values to obtain ΔC_t_ values (= C_t_ gene of interest − C_t_ of reference gene) using actin (ACTA; for endothelial cells) or ribosomal protein lateral stalk subunit P0 (RPLP0; for adipose tissue). To compare the expression pattern of genes in End.LepR-KO mice to WT mice, 2^−ΔΔCt^ values were calculated and expressed as -fold change to wild type mice (= ΔC_t_ of End.LepR-KO/ΔC_t_ of End.LepR-WT). The primer sequences used for real-time PCR were: LepR short isoform: for—GAA GTC TCT CAT GAC CAC TAC AGA TGA and rev—TTG TTT CCC TCC ATC AAA ATG TAA; LepR long isoform: for—GCA TGC AGA ATC AGT GAT ATT TGG and rev—CAA GCT GTA TCG ACA CTG ATT TCT TC; fatty acid binding protein-4 (*Fabp4*): for—GAT GCC TTT GTG GGA ACC TGG and rev—TTC ATC GAA TTC CAC GCC CAG; catenin beta-1 (*Ctnnb1*): for—TTA AAC TCC TGC ACC CAC CAT and rev—AGG GCA AGG TTT CGA ATC AA; *Ccnd1*; for—GTT CGT GGC CTC TAA GAT GAA GGA and rev—CAC TTG AGC TTG TTC ACC AGA AGC; perilipin-1 (*Plin1*): for—CTT TCT CGA CAC ACC ATG CAA ACC and rev—CCA CGT TAT CCG TAA CAC CCT TCA; leptin (*Lep*): for—GGA TCA GGT TTT GTG GTG CT and rev—TTG TGG CCC ATA AAG TCC TC; *Pdgfra*: for—TAT CCT CCC AAA CGA GAA TGA GA and rev—GTG GTT GTA GTA GCA AGT GTA CC; *Pparg*: for—CTCACAATGCCATCAGGTTT and rev—CTC TTG CAC GGC TTT CTA CGG; *Pref1*: for—CGT GAT CAA TGG TTC TCC CT and rev—AGG GGT ACA GCT GTT GGT TG; glucose transporter member 1 (*Glut1*): for—GCA GTT CGG CTA TAA CAC TGG and rev—GCG GTG GTT CCA TGT TTG ATT G; or isocitrate dehydrogenase (*Idh*): for—GGA GAA GCC GGT AGT GGA GA and rev—GGT CTG GTC ACG GTT TGG A; or sirtuin-3 (*Sirt3*): for—ATC CCG GAC TTC AGA TCC CC and rev—CAA CAT GAA AAA GGG CTT GGG; *Nos3*: for—GAC CCT CAC CGC TAC AAC AT and rev—CTG GCC TTC TGC TCA TTT TC; *Sele*: for—ATG CCT CGC GCT TTC TCT C and rev—GTA GTC CCG CTG ACA GTA TGC; and *Vcam1*: for—CTT CAT CCC CAC CAT TGA AG and rev—TGA GCA GGT CAG GTT CAC AG.

### Flow cytometry analysis

Freshly harvested VAT was transferred to ice-cold PBS, minced and digested in collagenase solution (1 mg/mL collagenase II in RPMI medium) at 37 °C for 1 h with constant shaking (ThermoMixer^®^ C; Eppendorf). After 1 h, the tissue was passed through a 70 μm cell strainer (greiner bio-one) to remove undigested cell debris and washed with FACS buffer (1% FBS, 2 mM EDTA in PBS) to collect the adipocytes. An equal number of cells were incubated for 10 min with unlabeled monoclonal antibody (mAb) against CD16/CD32 (eBioscience) to block nonspecific Fc receptor-mediated binding. Monocyte and macrophages were stained for 30 min with allophycocyanin (APC)-eFluor-780 or BV/11-labeled anti-mouse CD45 and Fixable Viability Dye eFluor506, APC-labeled anti-mouse CD11b, PE-labeled anti-mouse CD115, PerCP-Cy5.5-labeled anti-mouse Ly6C, BV650-labeled anti-mouse CD11c, Pacific Blue-labeled anti-mouse F4/80, fluorescein isothiocyanate-labeled anti-mouse CX3CR1, and APC-Cy7-labeled anti-mouse major histocompatibility complex (MHC) class II. T cells, B cells, NK cells, and polymorphonuclear leukocytes were excluded by staining with PE-Cy7-labeled anti-B220, anti-CD3, anti-NK1.1, and Ly6G antibodies (all from BioLegend). All flow cytometry measurements were performed on a BD LSR II (Becton Dickinson, Heidelberg, Germany). Flow Jo Version 10 was used for data analysis.

### Statistical analysis

Quantitative data are presented as mean ± standard deviation. Normal distribution was examined using the Shapiro–Wilk normality test. Differences between two groups were tested by Student’s *t* test for unpaired means or Mann–Whitney test, if normal distribution was not confirmed. If more than two groups were compared, One-Way or Two-Way Analysis of Variance (ANOVA) followed by Sidak’s Multiple Comparison test was performed if values were normally distributed, or Kruskal–Wallis test followed by Dunn’s multiple comparisons test, if not. For the comparison of two groups at different time points, Two-Way ANOVA was employed. Statistical significance was assumed if P reached a value < 0.05. All analyses were performed using GraphPad PRISM data analysis software (version 9.0 for Windows; GraphPad Software Inc.).

## Supplementary Information

Supplementary Figures.

Supplementary Information.

## Data Availability

The data that support the findings of this study are available from the corresponding author upon reasonable request.
