# Supplementary material for: Deletion of endothelial leptin receptors in mice promotes diet-induced obesity
Source: Sci Rep. 2023 May 22;13:8276. doi: 10.1038/s41598-023-35281-7 (PMC10203363; doi:10.1038/s41598-023-35281-7)
Supplement: Supplementary file 1 — Supplementary Figures. [file 41598_2023_35281_MOESM1_ESM.pdf]

Suppl. Figure 1

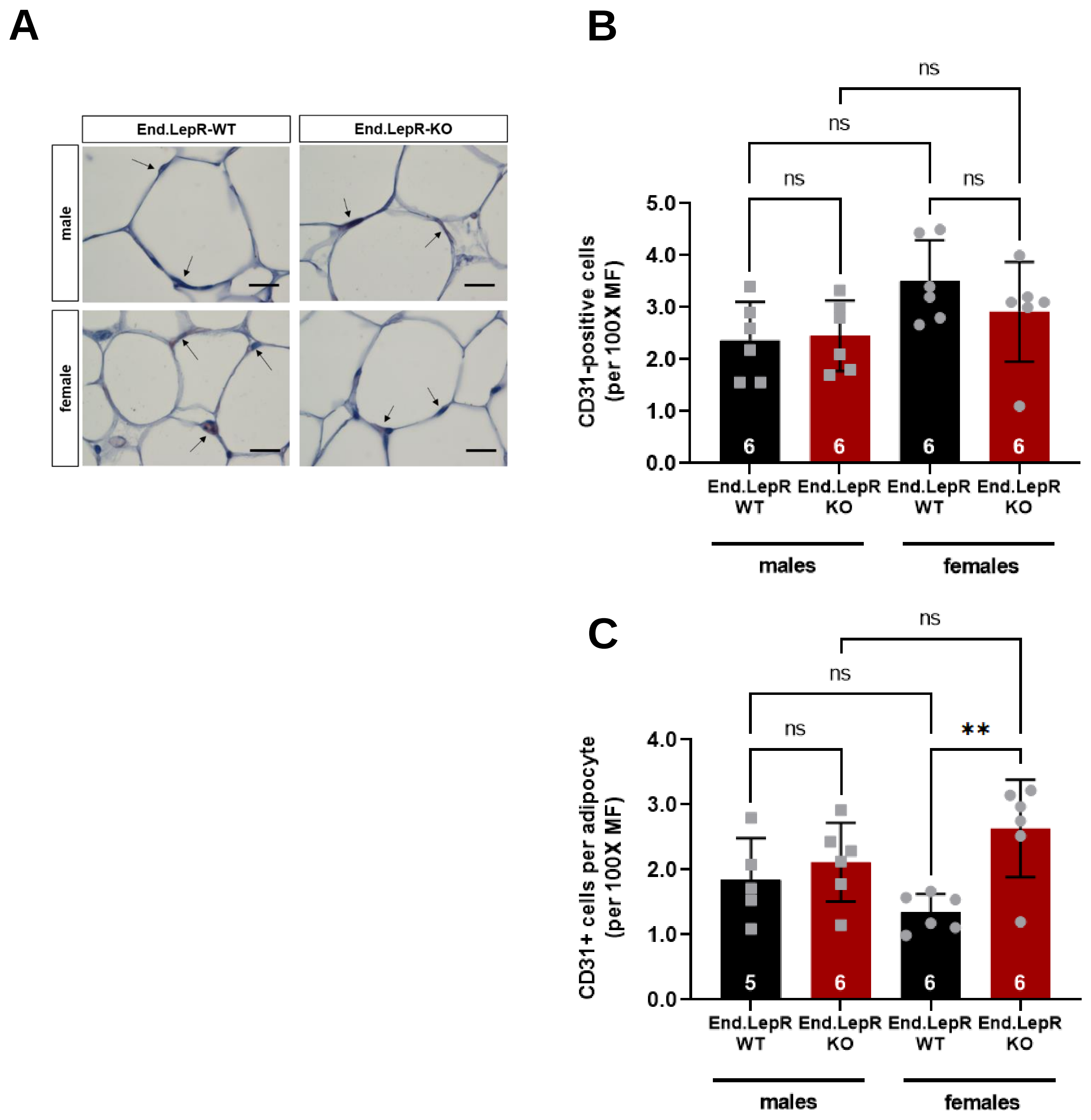

Supplemental Figure S1. Visceral adipose tissue vascularization. A, Representative microscopic photographs after CD31 immunohistochemical staining of cross-sections through the visceral adipose tissue of male (upper row) and female (lower row) End.LepR-WT and End.LepR-KO mice (n=6 each group) fed 45% high-fat diet for 16 weeks. Scale bars represent 10 µm. Quantitative analysis of the total (**B**) and relative (per adipocyte, **C**) number of CD31-positive cells per microscope field (MF) at 100X magnification. Data were analyzed using Kruskall-Wallis test followed by Dunn' multiple comparisons test. Ns, non-significant.

Suppl. Figure 2

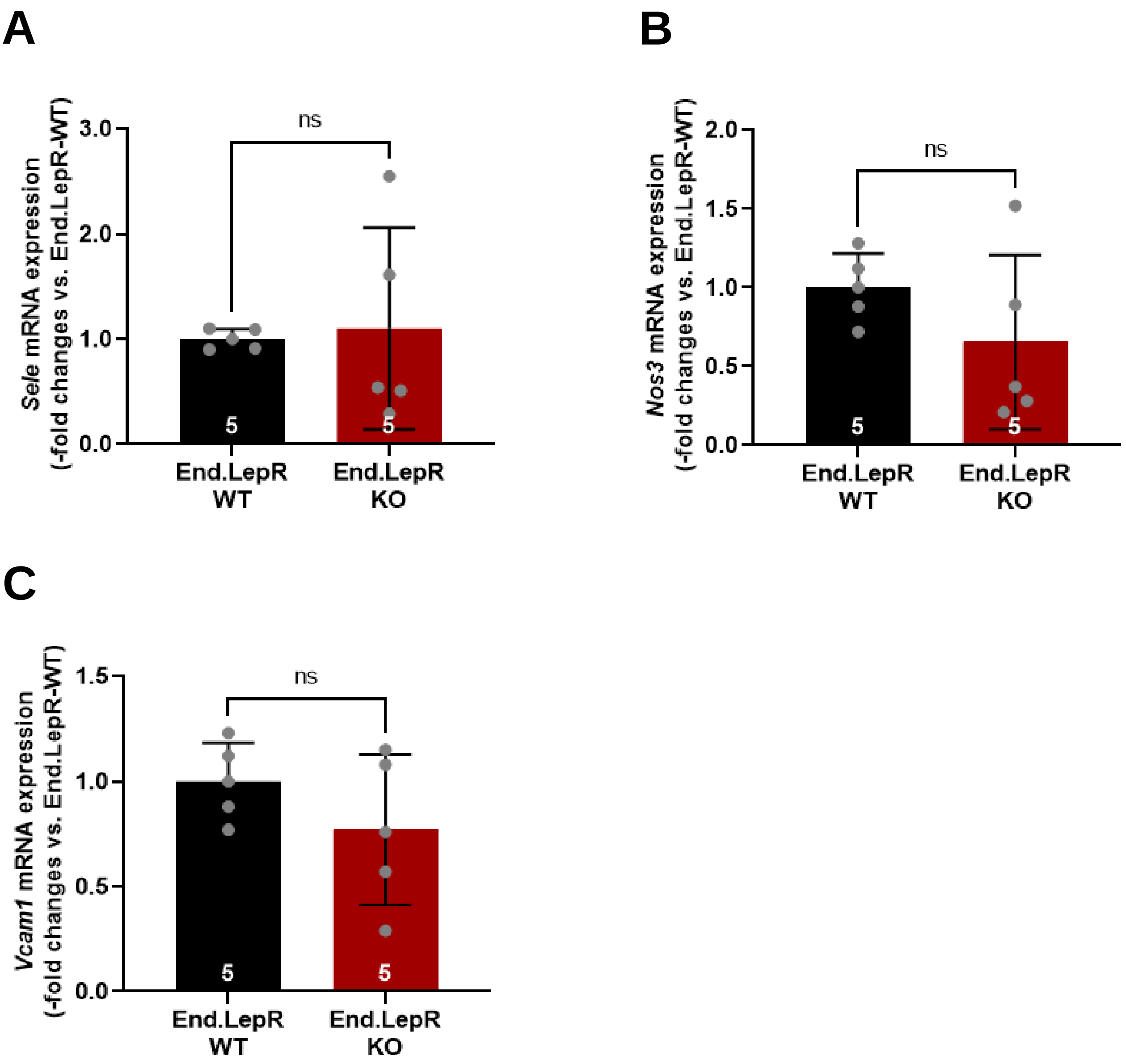

Supplemental Figure S2. Visceral adipose tissue endothelial gene expression. Quantitative real-time PCR analysis of primary endothelial cells isolated from visceral adipose tisse of female End.LepR-WT and End.LepR-KO mice (n=5 biological replicates per group) was performed to examine mRNA levels of E-selectin (*Sele*; **A**), endothelial nitric oxide synthase (*Nos3*; **B**) and vascular cell adhesion molecule-1 (*Vcam1*; **C**). Ns, non-significant (Student's t-test).

Suppl. Figure 3

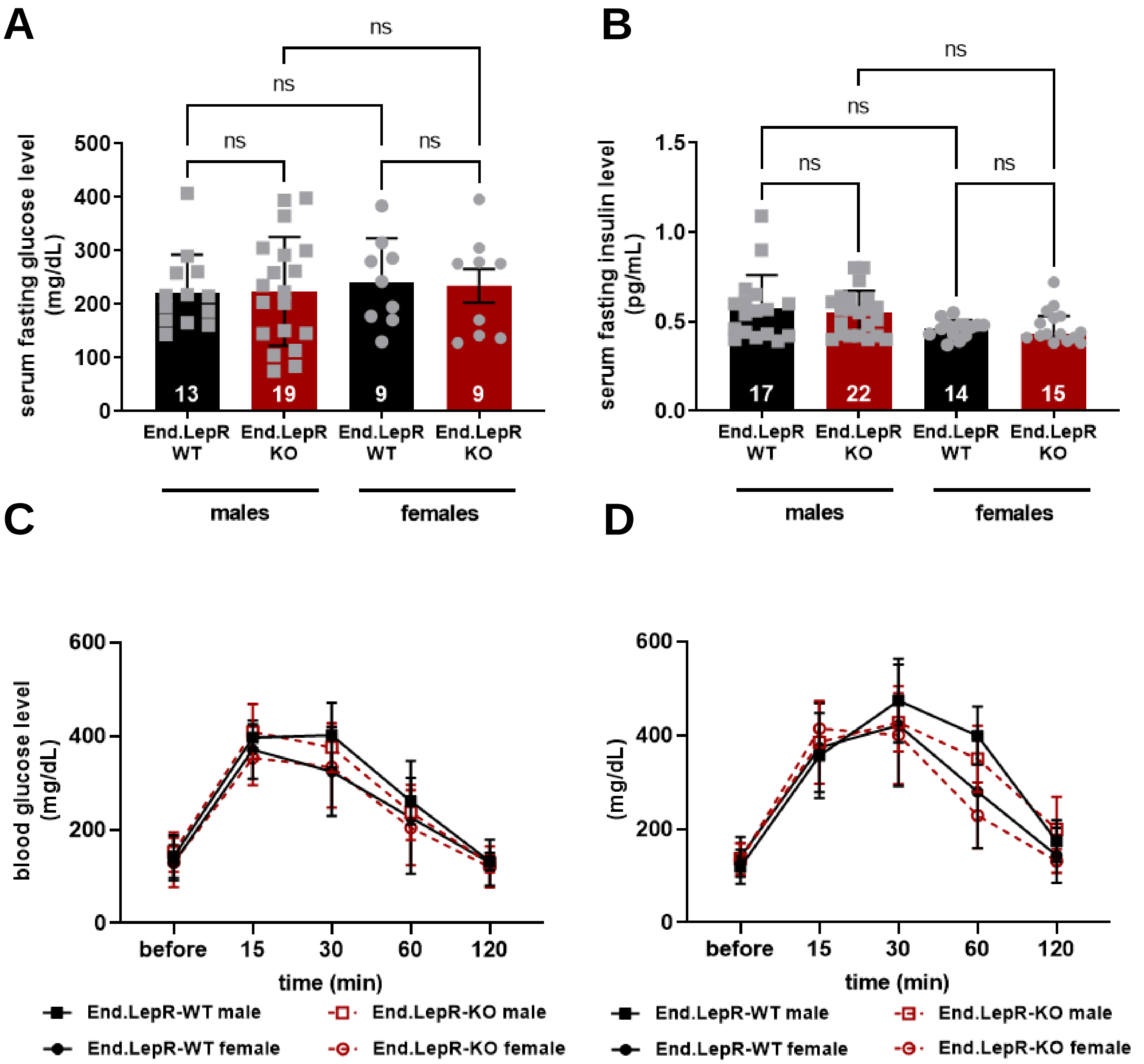

Supplemental Figure S3. Metabolic serum parameter. Fasting glucose (**A**) and insulin (**B**) levels in serum of male and female End.LepR-WT and End.LepR-KO mice fed high fat diet for 16 weeks. The number of mice examined in each experiment is given in the graph. Data were analyzed using Kruskall-Wallis test, Dunn's multiple comparisons test. ns, non-significant. Glucose tolerance tests were performed in male (n=14 WT and n=15 KO) and female (n=10 WT and n=12 KO) End.LepR-WT and End.LepR-KO mice, fed normal chow (**C**) or high fat diet (**D**) for 16 weeks. Mice were fasted for 6 hours followed by intraperitoneal injection of a 20% glucose solution (at a dosage of 2 g/kg body weight in 200 µL sterile NaCl). Data were analyzed using Two-Way ANOVA followed by Tukey's multiple comparions test. Non-significant differences are not shown.

Suppl. Figure 4

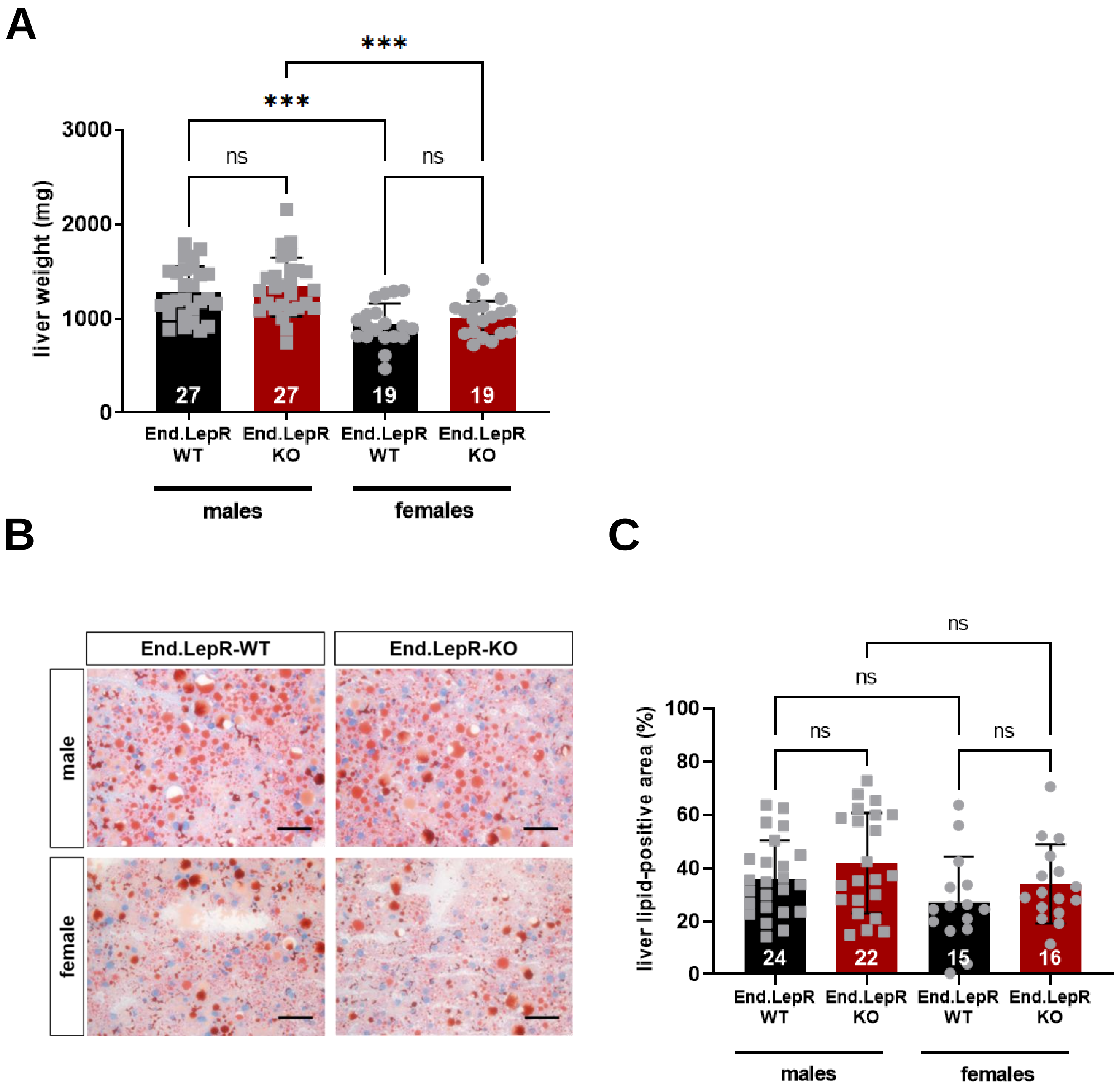

Supplemental Figure S4. Hepatic lipid accumulation in obesity. A, Liver wet weights of male and female End.LepR-WT and End.LepR-KO mice fed high-fat diet for 16 weeks. Data were analyzed using One-Way ANOVA, Sidak's multiple comparisons test. ns, non-significant. Oil red O staining of cryo-preserved tissue sections through the liver of male and female End.LepR-WT and End.LepR-KO mice. Representative images (**B**) and the results after quantification of the Oil red O-positive area (% of total area at 20X magnification) using ImagePro Plus software (**C**). Scale bars in B represent 20 µm. The number of mice examined per group is given in the graphs. Data were analyzed using One-Way ANOVA, Sidak's multiple comparisons test. ns, non-significant.

Suppl. Figure 5

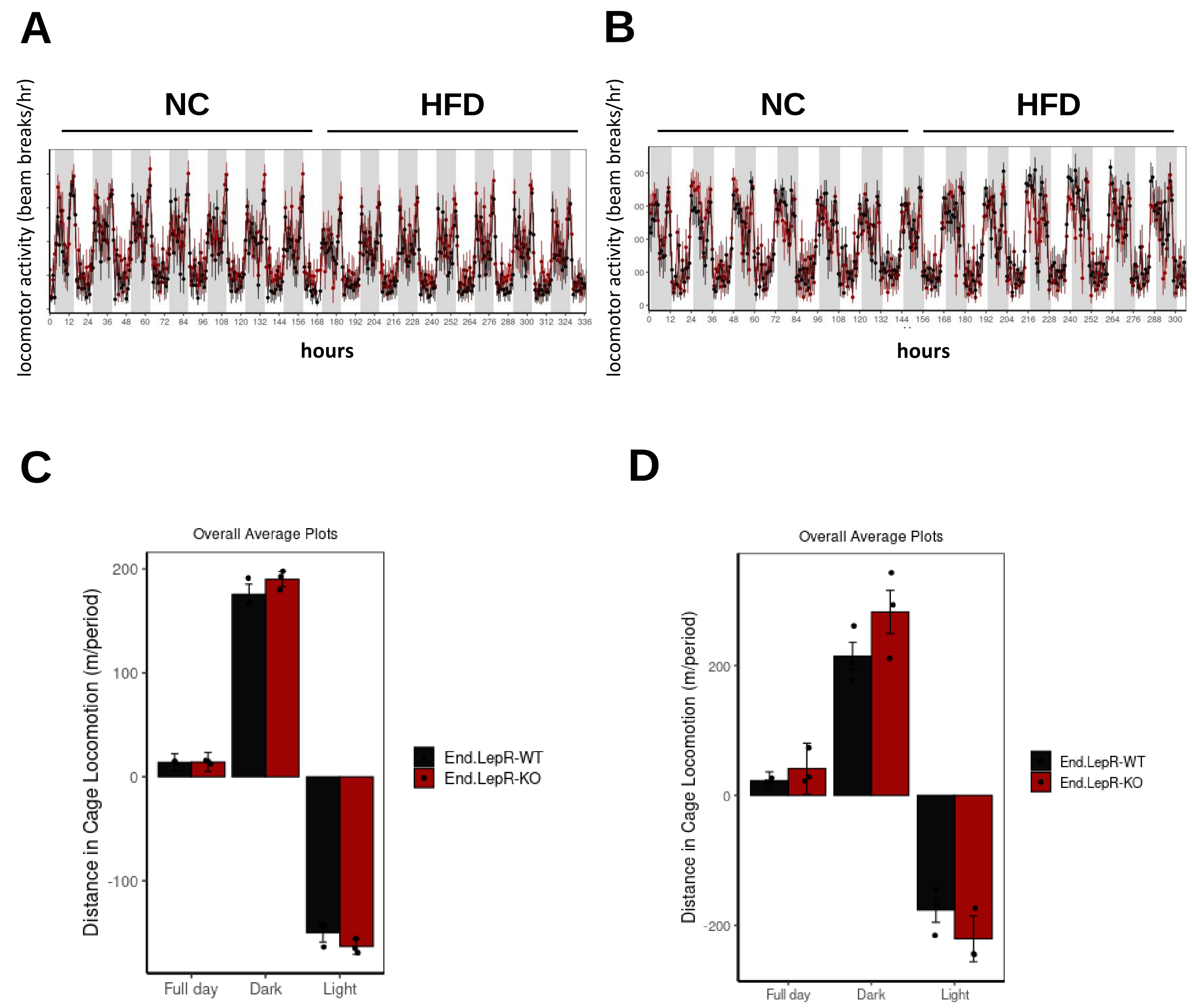

Supplemental Figure S5. Physical activity levels. Locomotor activity of male (**A**) and female (**B**) End.LepR-WT (black lines) and End.LepR-KO (red lines) during 7 days on normal chow (NC) followed by a switch to high fat diet (HFD). Distance in cage locomotion of male (**C**) and female (**D**) End.LepR-WT and End.LepR-KO mice. Data are shown for the full day or separately for either the dark and the light cycle. N=3 male and female mice, housed in indirect calorimetry cages, were examined genotype.

Suppl. Figure 6

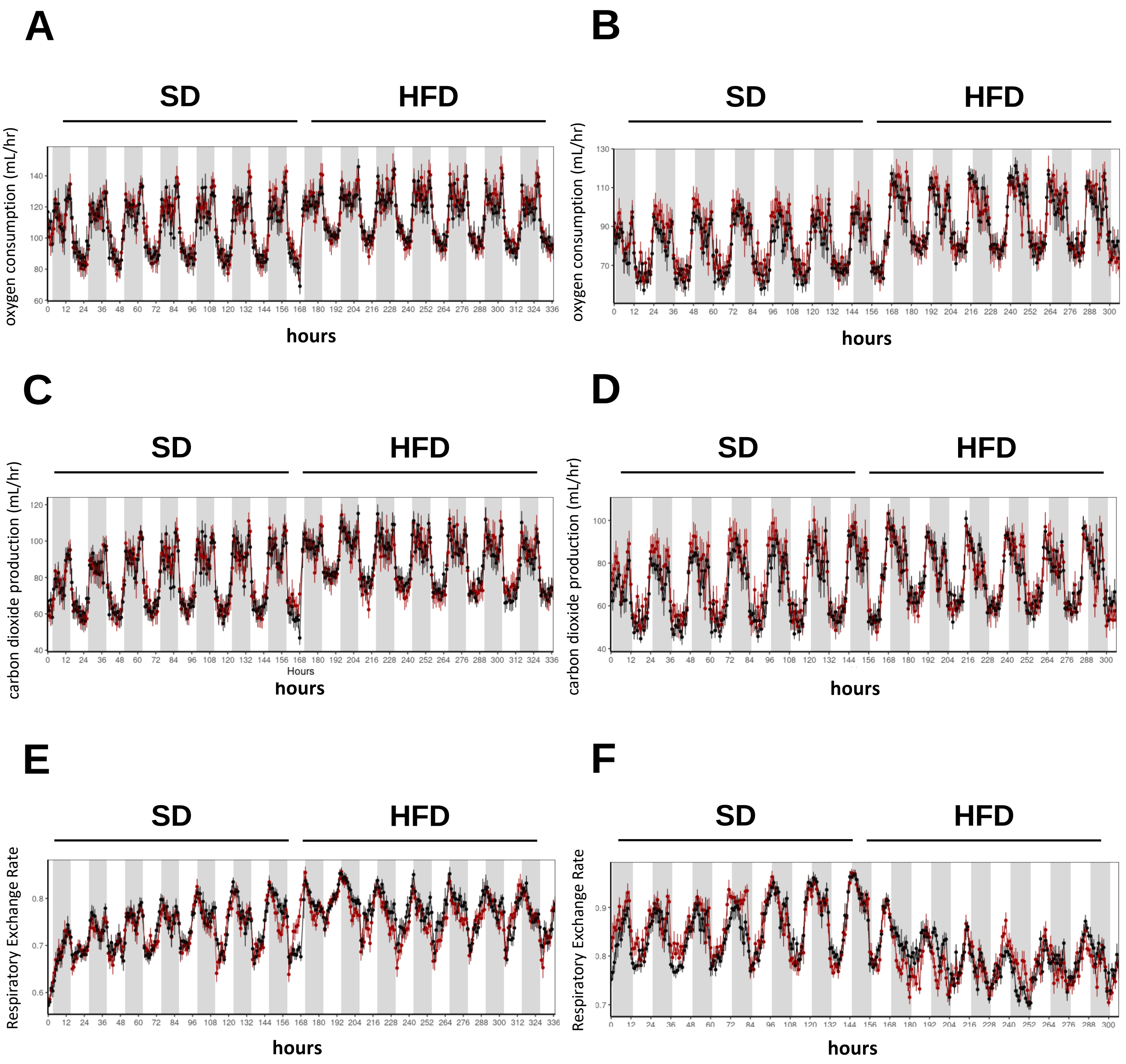

Supplemental Figure S6. Whole body energy consumption. Metabolic oxygen consumption (**A, B**), carbon dioxide release (**C, D**) and the respiratory exchange ratio (**E, F**) in male and female End.LepR-WT (black lines) and End.LepR-KO (red lines) mice during 7 days on standard laboratory diet (SD) followed by a switch to high fat diet (HFD) for 7 days are shown. N=3 male and female mice, housed in indirect calorimetry cages, were examined per genotype.

Suppl. Figure 7

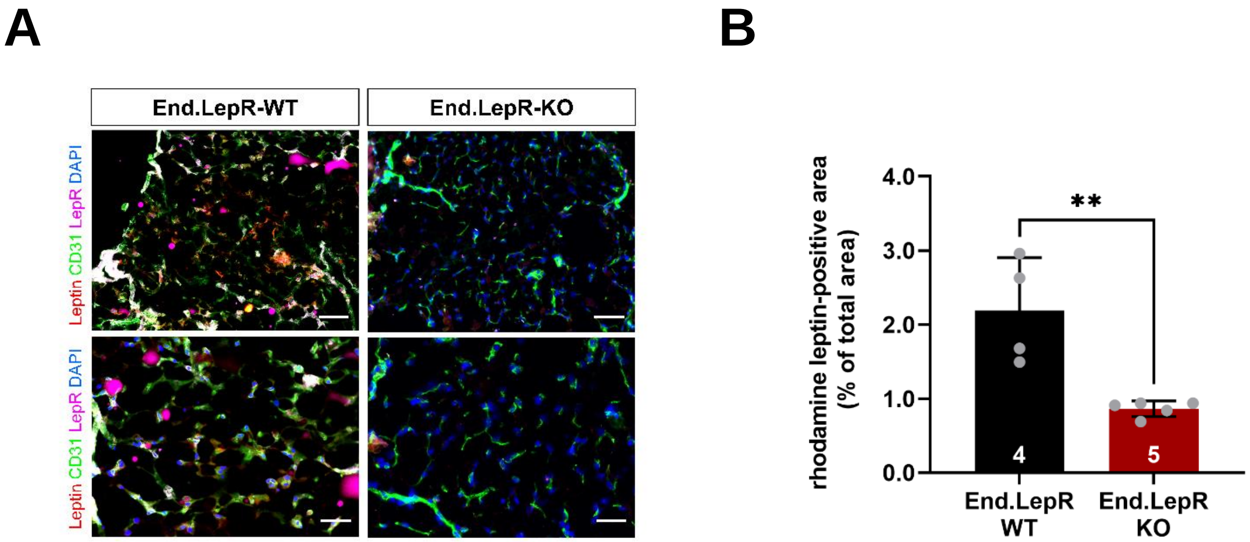

Supplemental Figure S7. Transport of exogenous recombinant leptin into visceral adipose tissue. Representative fluorescence microscopy images of cryo-embedded visceral adipose tissue (VAT) sections of End.LepR-WT and End.LepR-KO mice, i.p. injected with rhodamine-labeled leptin (red) and i.c. injected with FITC-labeled lectin to visualize endothelial cells (green), and immunostained for LepR (magenta). DAPI-positive cell nuclei are blue (**A**). Summary of the quantitative analysis of the rhodamine leptin-positive area per total area of 40X microscopic fields (**B**). **p=0.004, as determined using Student's t-test. Scale bars in A represent 20 µm (upper row) and 10 µm (lower row).

Suppl. Figure 8

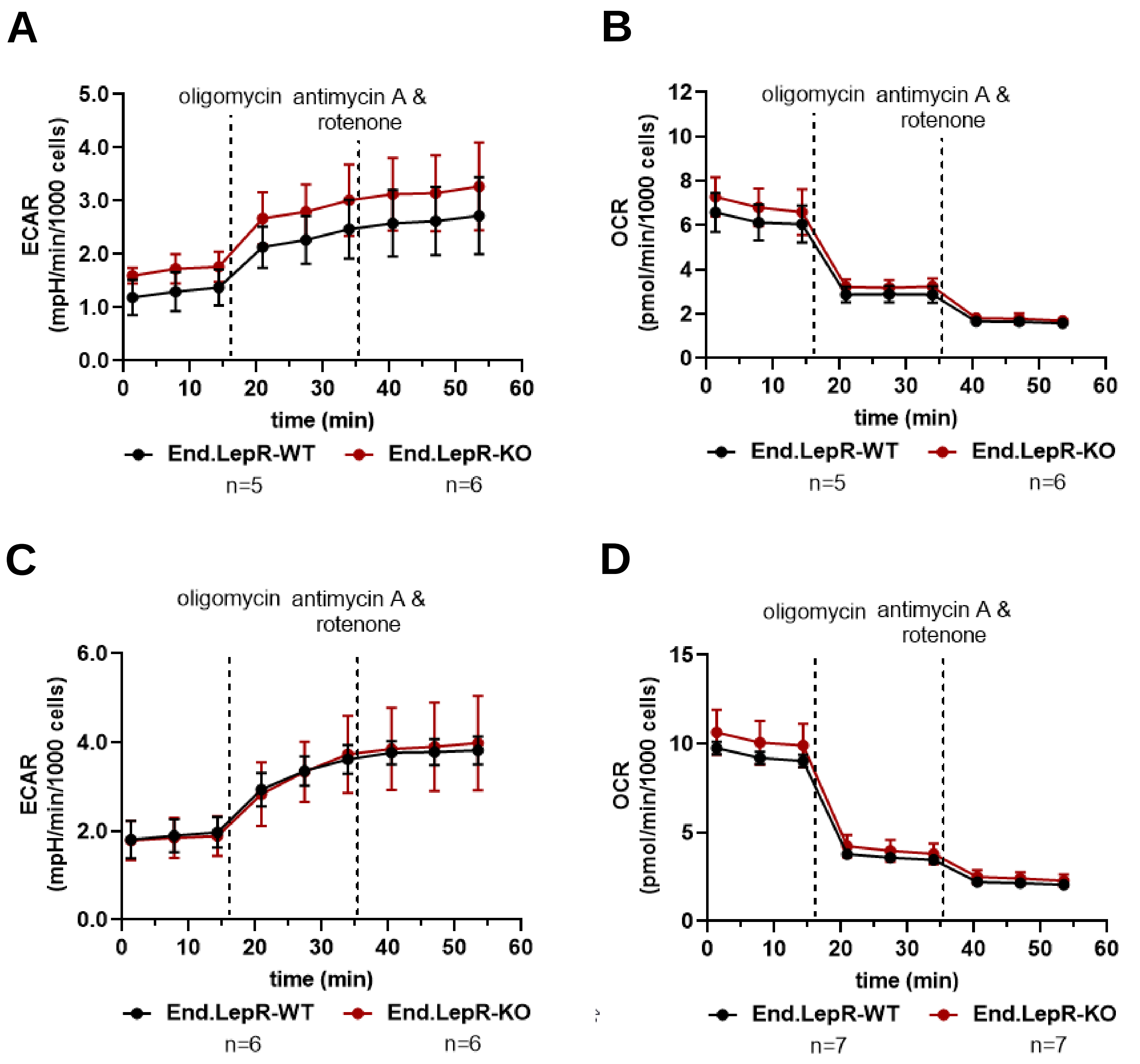

Supplemental Figure S8. Energy metabolism in primary brain and VAT endothelial cells. Representative examples of the extracellular acidification rate (ECAR; **A, C**) and the oxygen consumption rate (OCR; **B, D**) in live primary brain (A, B) and VAT (C, D) endothelial cells isolated from female End.LepR-WT and End.LepR-KO mice fed standard diet. Data were analyzed using Two-Way ANOVA, Sidak’s multiple comparison tests and did not show significant differences between the two genotypes.
