# Supplementary figures and images for: Deletion of endothelial leptin receptors in mice promotes diet-induced obesity
Source: Sci Rep. 2023 May 22;13:8276. doi: 10.1038/s41598-023-35281-7 (PMC10203363; doi:10.1038/s41598-023-35281-7)

Figure 1

A

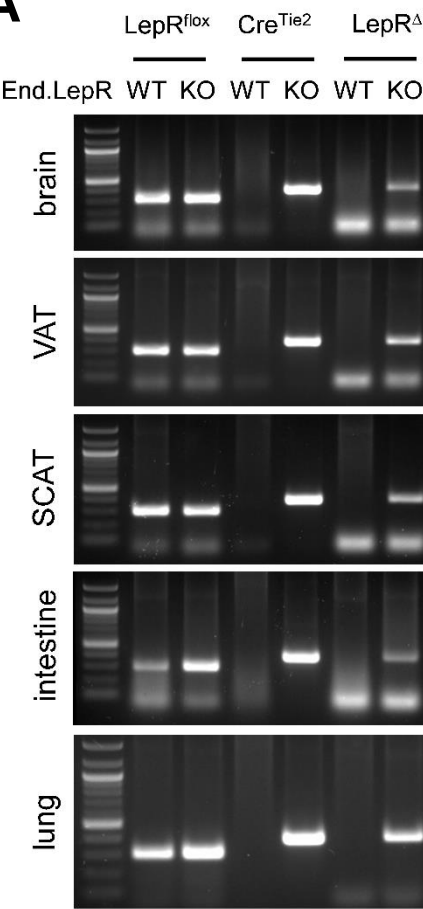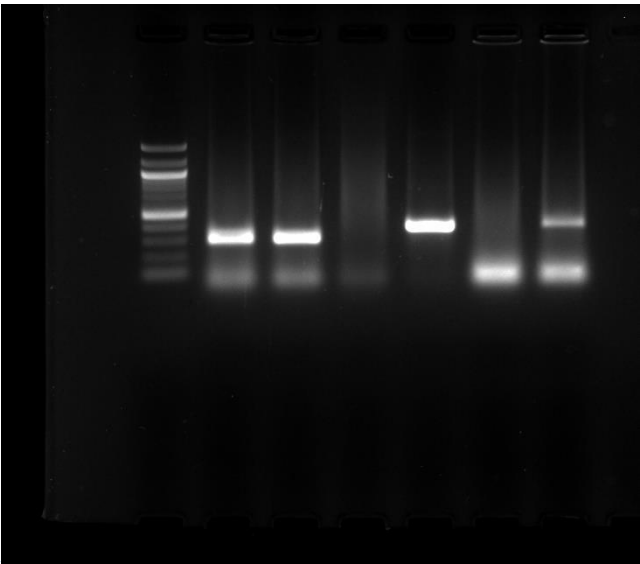

Figure 1

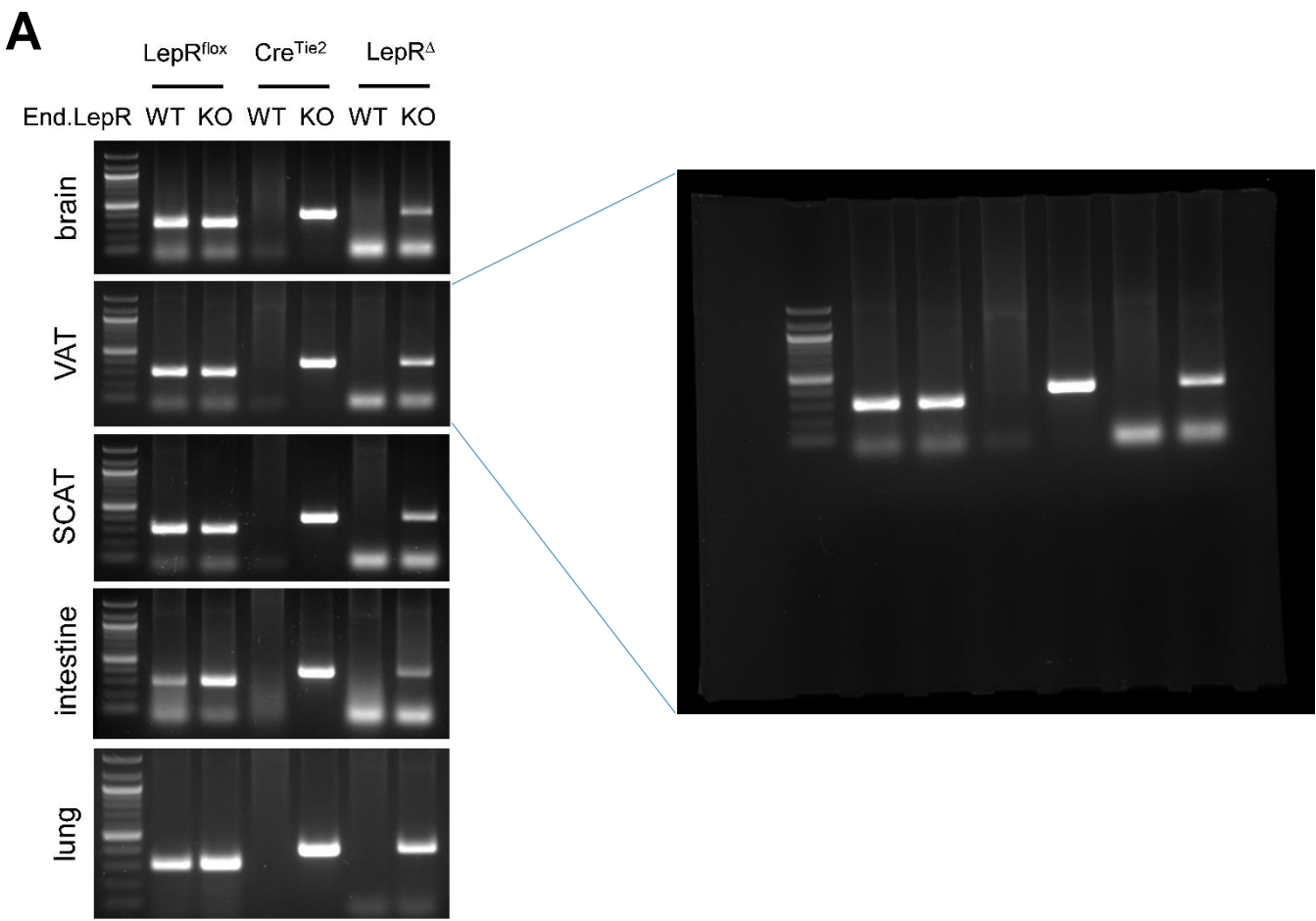

Figure 1

A

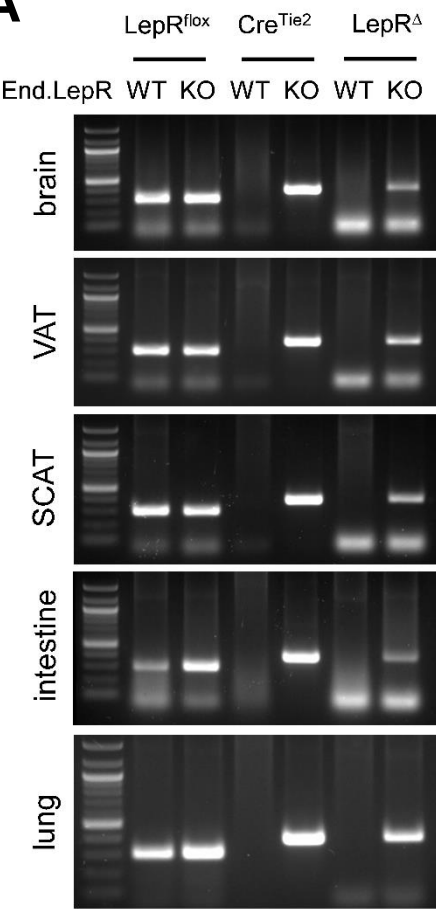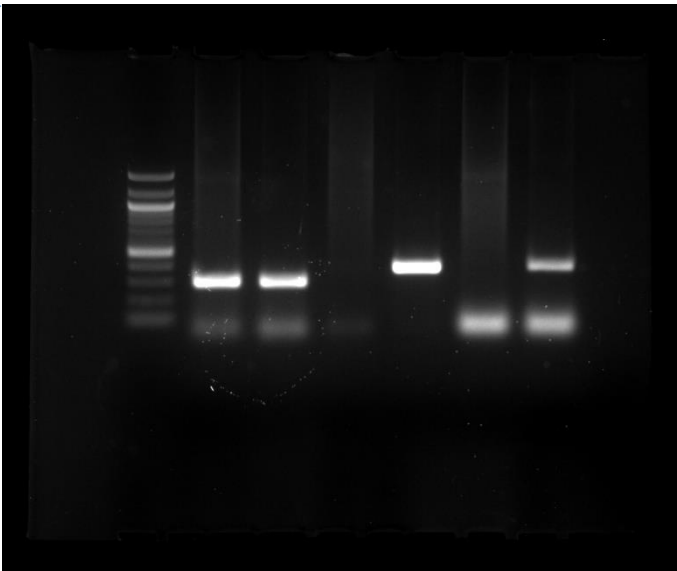

Figure 1

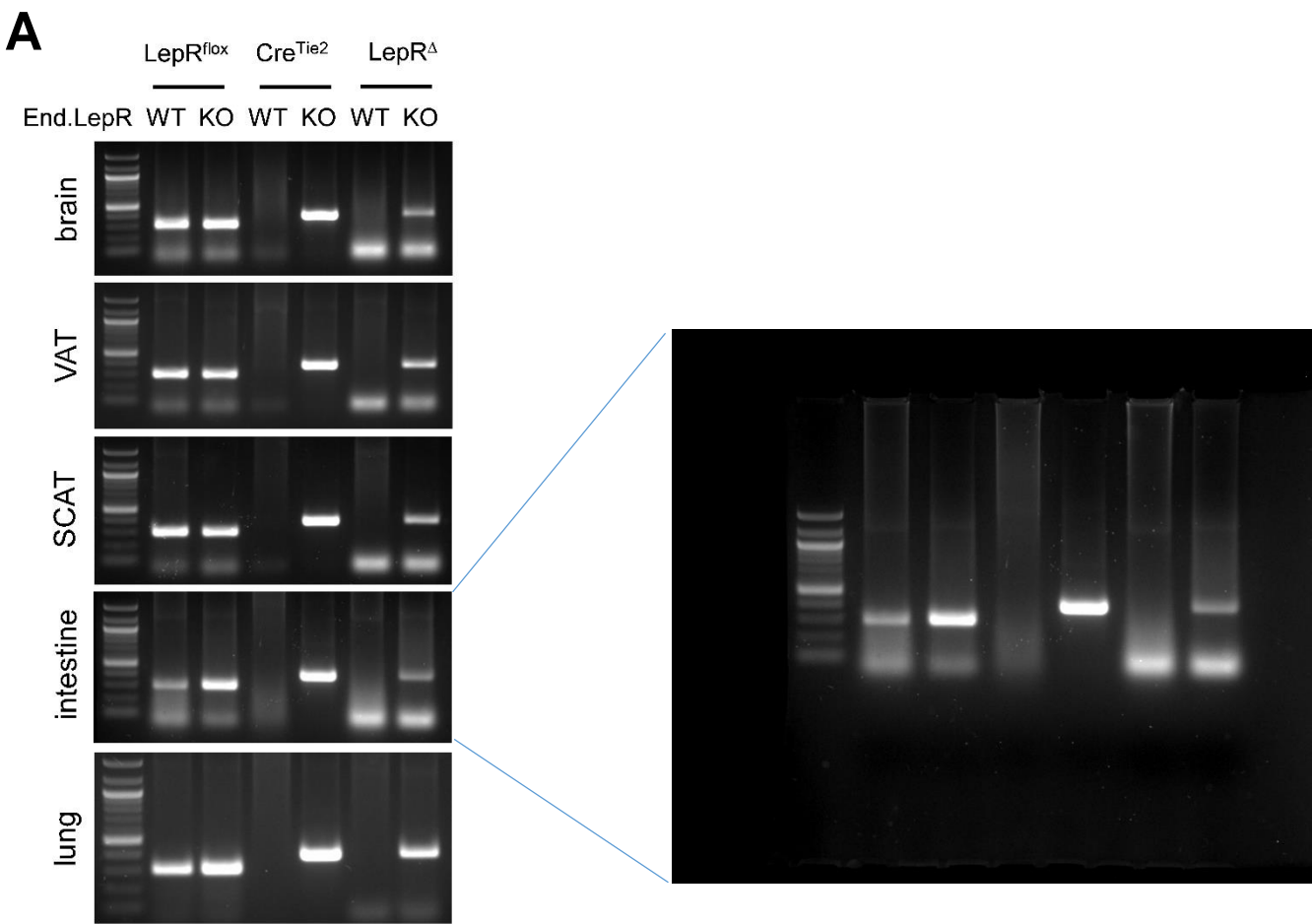

Figure 1

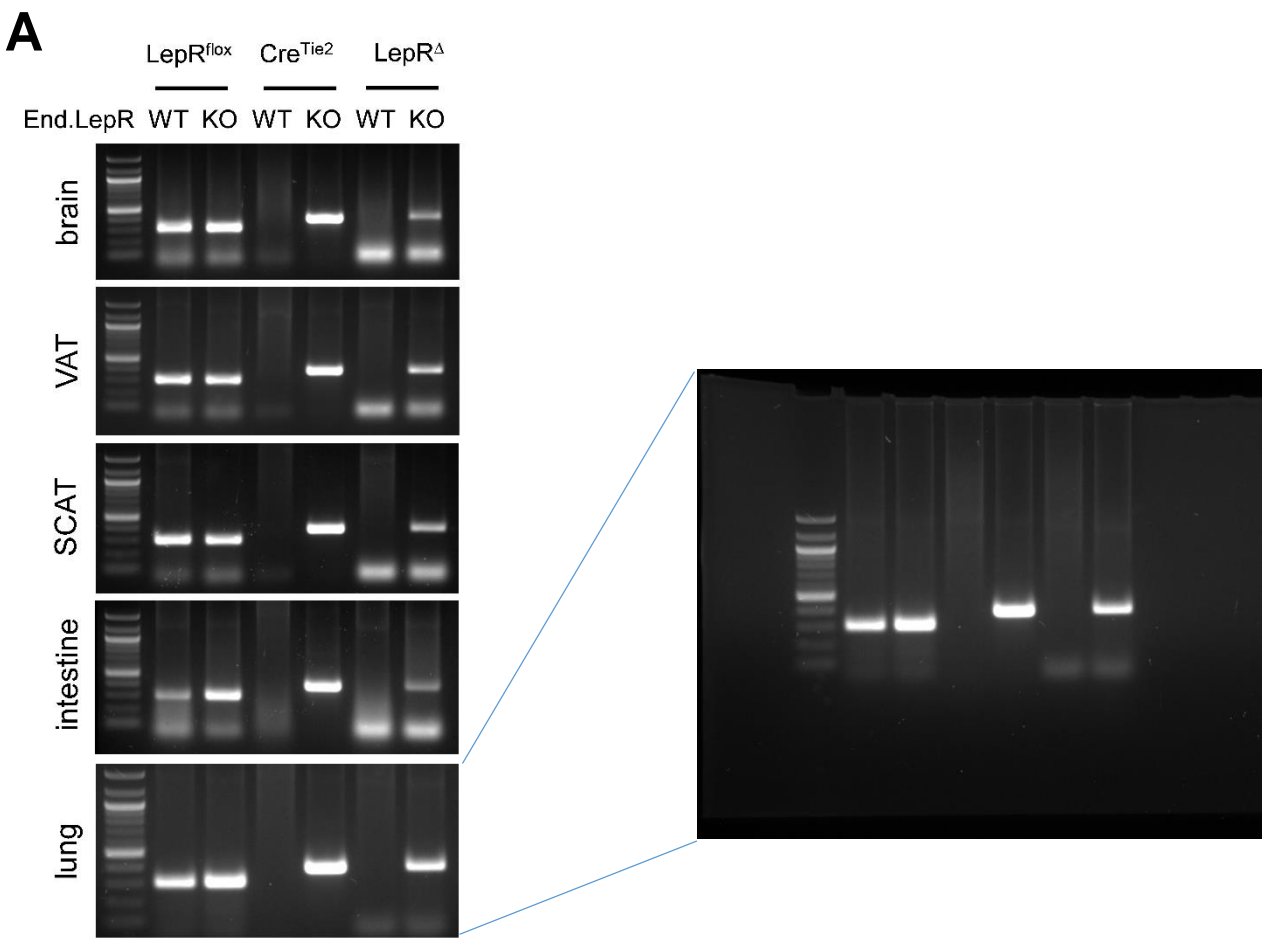

Supplement: Supplementary file 2 — Supplementary Information. [file 41598_2023_35281_MOESM2_ESM.pdf]
